# DEA-IDS: Drift-Aware Feature Selection and Few-Shot Adaptation for Cross-Domain IoT–IoMT Intrusion Detection

**DOI:** 10.3390/s26196200

**Published:** 2026-09-30

**Authors:** Büşra Günay, Mehmet Yavuz Yağcı

**Affiliations:** 1 Department of Computer Engineering, Faculty of Engineering and Natural Sciences, Istanbul Atlas University, 34408 Istanbul, Türkiye; 2 Department of Computer Engineering, Faculty of Engineering, Istanbul University-Cerrahpaşa, 34320 Istanbul, Türkiye

**Keywords:** intrusion detection systems (IDS), Internet of Medical Things (IoMT), cross-domain intrusion detection, domain shift, explainable artificial intelligence (XAI), drift analysis, stable feature selection, few-shot learning

## Abstract

Intrusion Detection Systems (IDSs) are essential for securing Internet of Things (IoT) and Internet of Medical Things (IoMT) environments, yet most machine learning-based IDSs assume that training and testing data follow similar distributions. In practice, domain shifts arising from differences in device characteristics, communication protocols, and traffic patterns can substantially increase false positive rates (FPRs), reducing operational reliability. This study proposes DEA-IDS (Drift-aware, Explainable and Adaptive Intrusion Detection System), a unified framework integrating SHAP-based explainability, statistical drift analysis via the Kolmogorov–Smirnov statistic and Wasserstein distance, drift-aware stable feature selection, and few-shot adaptation, evaluated on a CICIoT2023-to-CICIoMT2024 cross-domain transfer scenario. Under a leakage-free protocol in which drift statistics and few-shot samples are drawn exclusively from the target training split, DEA-IDS reduces FPR from 0.5468 to 0.0004 while maintaining an F1-score of 0.9944; threshold-, sample-size-, and feature-selection-control sensitivity analyses confirm this reduction reflects drift-aware stable feature selection rather than test-set leakage or dimensionality reduction alone. A per-attack-family analysis shows this improvement is concentrated in high-volume flood-style attacks and is accompanied by reduced detection of ARP spoofing, malformed-MQTT, and reconnaissance traffic, reported here as an explicit limitation. These results demonstrate that explicitly modeling feature stability before adaptation improves operational robustness for cross-domain intrusion detection in heterogeneous IoT–IoMT environments.

## 1. Introduction

Internet of Things (IoT) technologies have been increasingly integrated into healthcare systems through connected sensors, networked devices, and data-driven monitoring services [1,2]. This healthcare-oriented extension of IoT is commonly referred to as the Internet of Medical Things (IoMT). Wearable health sensors, remote patient-monitoring systems, smart medical devices, and connected diagnostic equipment are among the core components of IoMT infrastructures [1,2]. Although these systems support more accessible and efficient healthcare delivery, their reliance on heterogeneous devices, wireless communication, and continuous data exchange also exposes them to cybersecurity risks [2]. Security incidents in IoMT environments may compromise data confidentiality and system integrity and, in safety-critical settings, affect patient safety and continuity of care [3].

Intrusion Detection Systems (IDSs) provide an important complementary defence by identifying malicious or anomalous activities that bypass preventive security controls. Machine learning (ML)- and deep learning (DL)-based IDS approaches have been widely investigated because they can learn attack-related patterns from large volumes of network traffic [3,4]. However, IDS performance is commonly evaluated within a single dataset, where the training and test partitions originate from the same or highly similar data distributions. Consequently, the generalization capability of these models under different deployment environments remains insufficiently understood.

In operational deployment, the target network may differ from the training environment in terms of device types, communication protocols, traffic composition, and benign activity patterns. These differences may produce substantial changes in the underlying feature distributions. In this study, this source–target distribution mismatch is referred to as domain shift [5]. If patterns learned in the source domain do not adequately represent benign traffic in the target domain, legitimate samples may be classified as malicious, potentially increasing the false positive rate. In safety-critical environments such as healthcare, a high false-alarm burden can increase analyst workload and reduce the operational usability of an IDS [6]. Compared with conventional within-dataset experiments, cross-domain evaluations remain less frequently reported in IDS research. Accuracy, Recall, and F1-score are routinely presented, whereas the operational implications of false-positive predictions are not always examined in comparable detail [4,5].

Cross-domain IDS studies also do not consistently quantify source–target distribution differences at the individual-feature level. In parallel, explainable AI methods are commonly used to interpret model predictions or identify influential features after model training. Their joint use with statistical distribution measures to guide feature selection before adaptation remains comparatively underexplored [7]. Similarly, although few-shot adaptation approaches can utilize a limited amount of labeled target-domain data, the studies reviewed in this work do not explicitly combine limited target-domain adaptation with statistical filtering of distribution-sensitive features [8,9].

Based on these limitations, this study proposes DEA-IDS (Drift-aware, Explainable, and Adaptive Intrusion Detection System), an architecture designed to transfer an intrusion detection model trained in an IoT source domain to an IoMT target domain. The proposed framework integrates explainability analysis, statistical distribution measurement, stability-guided feature selection, and few-shot adaptation. It is evaluated using conventional classification metrics, with particular emphasis on the target-domain false positive rate.

The main contributions of this study are summarized as follows:

- We experimentally analyze the effects of domain shift in the IoT → IoMT transition from an operational reliability perspective.
- We investigate feature behavior through the combined use of SHAP-based explainability analysis and statistical distribution measurements.
- We develop a stability-guided feature selection mechanism and evaluate its effect under cross-domain conditions.
- We implement a few-shot adaptation strategy using a limited amount of labeled target-domain data.
- We evaluate the cross-domain robustness of DEA-IDS through FPR-focused operational analysis in addition to conventional metrics such as Accuracy and F1-score.
- We verify that our reported results are not an artifact of target-test-set information leakage into the drift-based feature-selection step by re-deriving all results under a leakage-free protocol, and we further establish their robustness through a KS-threshold sensitivity sweep, a five-seed few-shot sample-size sweep, comparisons against size-matched conventional feature-selection baselines, and an additional Stable-only ablation configuration that precisely attributes the observed FPR reduction to drift-aware stable feature selection.
- We disaggregate detection performance by attack family and show that the reported false-positive-rate reduction, while dominant for high-volume flood-style attacks, is accompanied by a substantial, previously unreported loss of detection for ARP spoofing, malformed-MQTT, and reconnaissance traffic, and we compare DEA-IDS against a target-only classifier and an unsupervised CORAL-based domain-adaptation baseline to further contextualize its operational trade-offs.

## 2. Related Works

As Internet of Things (IoT) deployments continue to expand across a wide range of application domains, protecting these interconnected environments from cyber threats has become increasingly important. Consequently, Intrusion Detection Systems (IDSs) have become a key component of IoT security architectures. The heterogeneous characteristics of IoT devices, their limited computational resources, and the continuously evolving threat landscape have encouraged extensive research on machine learning (ML)- and deep learning (DL)-based intrusion detection methods. Existing studies have investigated a broad range of learning paradigms, including conventional machine learning algorithms, feed-forward neural networks, deep convolutional architectures, and hybrid attention-based models, with the objective of improving detection performance while maintaining computational efficiency [10,11,12,13].

A common characteristic of the recent literature is the emphasis on improving classification performance through increasingly sophisticated model architectures. Comparative evaluations of multiple machine learning algorithms have demonstrated that carefully selected classifiers can achieve high detection performance with relatively low computational overhead [10]. Other studies have focused on lightweight neural network architectures to balance detection accuracy and resource efficiency in IoT environments [11], whereas deep learning approaches have further enhanced feature representation through convolutional networks and hierarchical learning mechanisms [12]. More recently, hybrid architectures integrating CNN, BiLSTM, and attention mechanisms have been proposed to capture both spatial and temporal traffic characteristics, yielding further improvements in attack classification performance [13].

Despite these advances, most existing IoT IDS studies share an important limitation. Although they report high detection performance, their evaluation protocols generally assume that training and testing data originate from the same or highly similar distributions, leaving their robustness under heterogeneous deployment environments largely unexplored. Consequently, the reported improvements are largely limited to in-distribution scenarios, while cross-domain generalization remains insufficiently investigated. Furthermore, evaluation is predominantly based on conventional metrics such as Accuracy, Precision, Recall, and F1-score, whereas operationally important indicators, particularly the False Positive Rate (FPR), receive considerably less attention. Since false alarms directly influence the workload of security analysts and the practical usability of IDS, these limitations indicate that improving classification accuracy alone is insufficient for practical IoT intrusion detection. Instead, intrusion detection systems should maintain reliable performance under distributional changes and heterogeneous deployment environments while minimizing operational false alarms.

### 2.1. IoMT Security and Intrusion Detection

The Internet of Medical Things (IoMT) has become an essential part of modern healthcare by interconnecting medical devices, monitoring systems, and clinical platforms to support continuous healthcare services. As these interconnected environments continue to expand, ensuring their security has become increasingly challenging because of the sensitivity of medical information, the heterogeneous nature of IoMT devices, and the strict reliability requirements of healthcare systems. Compared with conventional IoT deployments, IoMT environments require intrusion detection mechanisms that deliver not only high detection performance but also reliable and timely responses to cyber attacks that may disrupt healthcare services or compromise patient safety. Accordingly, developing dependable intrusion detection systems has become an active area of research for securing medical cyber–physical systems [14,15,16,17].

Recent IoMT intrusion detection studies have investigated a variety of machine learning and deep learning solutions. These include explainable neural-network models, hybrid feature-selection methods, lightweight security frameworks for healthcare environments, and domain-adaptation strategies intended to improve detection performance under IoMT-specific network conditions [14,15,16,17]. Collectively, these approaches demonstrate that combining advanced learning architectures with feature engineering and explainability can substantially enhance intrusion detection performance in specialized IoMT environments. In particular, explainable learning frameworks improve the interpretability of detection decisions, whereas hybrid feature selection and domain adaptation techniques contribute to more robust feature representations under medical network conditions [14,15,16].

Despite these advances, current IoMT IDS research remains largely focused on models developed and evaluated within the same medical domain. Most studies optimize detection performance using IoMT-specific datasets without explicitly investigating whether knowledge learned from conventional IoT environments can be effectively transferred to medical networks. As a result, the impact of distributional differences between IoT and IoMT traffic has received relatively limited attention. Furthermore, evaluation continues to emphasize traditional classification metrics, whereas the operational consequences of increased false alarms during cross-domain deployment are rarely analyzed. These observations indicate that future IoMT IDS should emphasize not only detection accuracy but also cross-domain robustness and operational reliability under heterogeneous deployment conditions.

### 2.2. Explainable Artificial Intelligence in Intrusion Detection Systems

The increasing use of machine learning (ML) and deep learning (DL) models in intrusion detection has also increased the need to better understand how these models produce their predictions. In this context, Explainable Artificial Intelligence (XAI) provides mechanisms for interpreting model behavior by identifying the contribution of individual input features to detection outcomes, thereby improving model transparency and supporting confidence in IDS decisions. Among existing explanation methods, SHAP and LIME are the techniques most frequently employed in IDS research because they can be applied to different learning models and provide both instance-level and model-level explanations of prediction behavior [18,19,20,21].

Recent studies have integrated XAI into IDS to improve model interpretability, support forensic analysis, and facilitate feature-level investigation of attack detection mechanisms. Existing research has proposed explainable IDS frameworks, XAI-assisted feature analysis, and comparative evaluations of SHAP and LIME for understanding classifier decisions across different intrusion detection scenarios [18,19,20]. These approaches demonstrate that explanation techniques can help security analysts identify influential network features, validate model behavior, and increase confidence in automated detection systems. More recently, SHAP-based analyses have also been explored for distinguishing different forms of distributional change (e.g., concept drift and scale drift) in network traffic, suggesting that explanation methods may provide valuable insights into data distribution characteristics beyond conventional model interpretation [21]. However, these studies primarily employ explainability to interpret model decisions rather than to analyze feature behavior under changing data distributions.

Despite these advances, most existing XAI-based IDS studies primarily employ explanation methods as post-hoc interpretation tools. Their main objective is to explain prediction outcomes or improve model transparency after training, rather than incorporating explainability into the analytical process itself. Consequently, XAI is rarely used to support drift analysis, assess feature stability across domains, or guide the development of intrusion detection models that remain reliable under changing data distributions. These limitations indicate that explainability should not only improve the interpretability of IDS decisions but also contribute to understanding how distributional changes affect feature behavior and operational reliability in cross-domain environments.

### 2.3. Cross-Domain Intrusion Detection

The increasing diversity of network environments has shifted the focus of intrusion detection research from conventional in-distribution evaluation toward cross-domain generalization. In practical deployments, IDS models are frequently required to operate in target environments that differ substantially from the networks on which they were originally trained. To address this challenge, recent studies have investigated transfer learning, domain adaptation, and cross-dataset learning strategies to improve knowledge transfer across heterogeneous network environments [22,23,24,25]. These approaches aim to reduce the performance degradation caused by distributional discrepancies while minimizing the amount of labeled data required in the target domain.

Existing cross-domain IDS research has demonstrated that transfer learning and domain adaptation can improve detection performance when source and target domains exhibit moderate distributional differences [22,23,24,25]. Several studies have explored feature alignment techniques, auxiliary-domain learning, and transferable feature representations to enhance model generalization across different datasets [23,24,25]. A related, database-oriented line of work has instead approached cross-dataset intrusion detection from a data-management perspective, proposing frameworks for combining and harmonizing heterogeneous intrusion-detection datasets so that they can be jointly used for training [26]; this complements the present study’s feature-level (rather than database-level) approach to establishing a common representation across the source and target domains (Section 4.1).

More recently, SHAP-based analyses have also been employed to investigate how feature importance changes after domain adaptation, providing additional insights into the transferability of learned representations. Nevertheless, these studies primarily utilize explainability to interpret adaptation outcomes rather than to identify stable features before model adaptation or to quantify feature-level distributional drift [22]. Collectively, these studies demonstrate that cross-domain learning can partially mitigate distribution mismatch across heterogeneous network environments.

Despite these advances, several important challenges remain unresolved. Most existing studies primarily evaluate improvements in terms of Accuracy, Precision, Recall, or F1-score, whereas the operational impact of domain shift on false positive rates is rarely examined. Moreover, although domain adaptation techniques attempt to reduce distribution mismatch, relatively few studies explicitly quantify feature-level drift or identify which features remain stable across different domains before model adaptation. Consequently, the relationship between distributional changes, feature stability, and operational reliability remains insufficiently understood. These limitations suggest that effective cross-domain intrusion detection should not only transfer learned knowledge between domains but also explicitly analyze distributional changes and preserve stable feature representations to maintain reliable performance under heterogeneous deployment conditions.

### 2.4. Domain Adaptation and Few-Shot Learning for Intrusion Detection Systems

Domain adaptation and few-shot learning are widely studied approaches for improving the ability of intrusion detection systems to operate across different network environments, particularly when only a limited amount of labeled target-domain data is available. Rather than requiring large annotated datasets for every deployment scenario, these methods exploit knowledge learned from a source domain and adapt it to a related target domain while minimizing the need for additional labeled samples. For this reason, few-shot learning has become an attractive approach for intrusion detection in heterogeneous environments where obtaining sufficient labeled network traffic is costly or impractical [9,27,28,29].

Recent studies have proposed various meta-learning, metric-learning, and transfer learning strategies to improve intrusion detection under limited supervision [19,20,21,22]. Existing approaches demonstrate that incorporating a small number of labeled target-domain samples can substantially improve detection performance compared with models trained exclusively on source-domain data. In particular, few-shot learning has been successfully applied to IoT intrusion detection by learning transferable representations that facilitate rapid adaptation to previously unseen attack scenarios and heterogeneous deployment environments [27,28,29]. These findings indicate that limited target-domain information can effectively support model adaptation while reducing annotation costs.

Despite these promising results, current few-shot IDS research primarily focuses on improving classification accuracy rather than understanding the underlying causes of cross-domain performance degradation. Most existing approaches implicitly assume that transferable feature representations can be learned directly through adaptation without explicitly evaluating which network features remain stable across different domains. Consequently, feature-level distributional changes and their relationship to operational metrics such as the False Positive Rate (FPR) remain largely unexplored. These limitations suggest that effective cross-domain intrusion detection requires not only limited target-domain adaptation but also systematic analysis of feature stability and distributional drift to ensure reliable operational performance under heterogeneous network conditions.

### 2.5. Research Gap and Positioning of DEA-IDS

The literature reviewed above demonstrates substantial progress in IoT and IoMT intrusion detection through advances in machine learning, explainable artificial intelligence, cross-domain learning, and few-shot adaptation [30,31,32]. Nevertheless, these research directions have largely evolved independently. Although XAI, domain adaptation, and few-shot learning have each demonstrated promising results, their integration into a unified framework that jointly analyzes feature behavior, quantifies distributional drift, and supports robust cross-domain adaptation remains limited. Existing studies predominantly emphasize improving classification performance using conventional metrics such as Accuracy, Precision, Recall, and F1-score, while the operational impact of domain shift remains comparatively underexplored [30,31,32]. In particular, relatively few studies explicitly investigate how distributional changes influence feature behavior, increase false positive rates, or affect operational reliability in heterogeneous deployment environments. Likewise, explainability techniques are primarily employed to interpret model decisions rather than to support drift analysis or evaluate feature stability across domains [33]. Similarly, transfer learning and few-shot learning approaches focus on improving model adaptation without systematically identifying which features remain reliable under changing data distributions [9,32,34]. Consequently, there remains limited research integrating explainability-assisted feature analysis, statistical drift quantification, stable feature selection, and limited target-domain adaptation into a unified framework for operationally reliable cross-domain intrusion detection. Motivated by these limitations, this study proposes DEA-IDS, a unified framework that combines SHAP-assisted feature analysis, statistical drift analysis, stable feature selection, and few-shot adaptation to improve operational robustness under cross-domain deployment. Rather than focusing solely on classification accuracy, DEA-IDS explicitly addresses feature instability and distributional changes to reduce false positive rates and maintain reliable intrusion detection performance in heterogeneous network environments.

We clarify the nature of this contribution explicitly: none of SHAP, the KS statistic, the Wasserstein distance, few-shot adaptation, or XGBoost is individually novel to this paper, and DEA-IDS does not introduce a new learning algorithm. The contribution instead lies in the specific integration and design choice of using drift statistics computed on the target domain before adaptation to actively filter the feature space (Section 3.4), rather than using explainability or drift analysis only as a post-hoc, descriptive tool, together with the systematic empirical demonstration—via the ablation, sensitivity, and control-baseline analyses in Section 5.2, Section 5.8, Section 5.9 and Section 5.10—that this specific design choice, and not dimensionality reduction or few-shot adaptation in general, is responsible for the observed operational-reliability improvement.

## 3. Proposed DEA-IDS Framework

### 3.1. DEA-IDS Architecture

**Figure 1:**
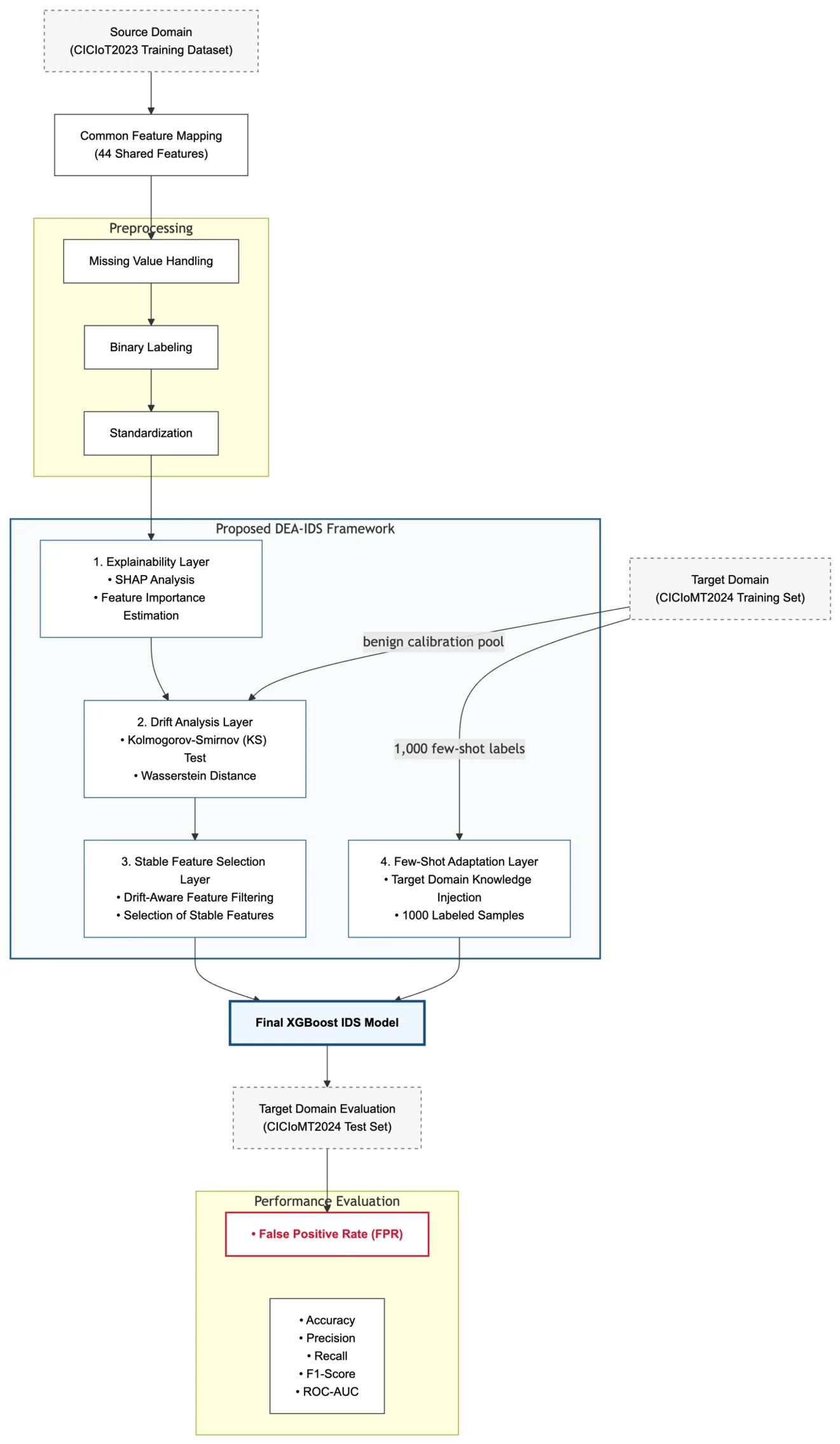
Overview of the proposed DEA-IDS framework integrating SHAP-based explainability, drift-aware feature selection, and few-shot adaptation for cross-domain IoT-to-IoMT intrusion detection. For clarity, target-domain (CICIoMT2024) data enters the pipeline at exactly two points: (i) the CICIoMT2024 *training* split contributes to the Drift Analysis layer’s statistics and supplies the labeled samples used by the Few-Shot Adaptation layer (Section 3.3 and Section 3.5); and (ii) the CICIoMT2024 *test* split, kept fully disjoint from (i), is used only at the final Evaluation stage. The Explainability (SHAP) layer is computed on held-out training subsamples from both domains (Section 3.2) and, like the source-domain preprocessing step, does not use the target test split. The diagram depicts both target-domain entry points explicitly: the trusted-benign calibration pool feeds the Drift Analysis layer, and the 1000-sample few-shot labels feed the Few-Shot Adaptation layer (Section 5.14 and Section 5.22).

The proposed DEA-IDS (Drift-aware, Explainable and Adaptive Intrusion Detection System) was developed to mitigate the domain shift problem that arises when an intrusion detection model trained in an IoT environment is transferred to a target environment with different characteristics, such as IoMT. DEA-IDS presents a multi-stage architecture that integrates explainability analysis, statistical drift measurement, stable feature selection, and few-shot adaptation within a unified framework. Figure 1 illustrates the overall workflow of DEA-IDS. The process begins by transforming the source-domain CICIoT2023 dataset into a common feature space and applying the preprocessing steps. During preprocessing, missing values are handled, binary labels are generated, and feature standardization is performed. The purpose of this stage is to establish a consistent feature representation shared by both the source and target domains. The next stage corresponds to the explainability layer, where SHAP analysis is employed to identify the key features influencing the model’s predictions. The resulting explanations not only facilitate the interpretation of model behavior but also provide complementary information for interpreting the drift analysis performed in the subsequent stage. In the second stage, distributional differences between the source and target domains are quantified using the Kolmogorov–Smirnov (KS) test and the Wasserstein distance. Based on the drift analysis results, features exhibiting more stable behavior across domains are identified and retained by the stable feature selection layer. This process aims to reduce the model’s dependence on features that undergo substantial distributional changes in the target domain. In the following stage, few-shot adaptation is performed using a limited number of labeled samples representing the target domain. In this mechanism, a limited set of labeled target-domain samples is incorporated into the source-domain training set, allowing the classifier to learn target-specific traffic characteristics during retraining. The final XGBoost model is trained using the stable feature subset together with an augmented training set created through a few-shot adaptation procedure. During the evaluation stage, the trained model is tested on the CICIoMT2024 test set, which serves as the target domain. The evaluation metrics and the complete experimental protocol are described in detail in Section 3.6. Although multiple performance metrics are reported, the False Positive Rate (FPR) is considered the primary evaluation criterion because of its direct impact on operational reliability. Rather than attempting to address the domain shift problem solely by employing more sophisticated classifiers, the main design principle of DEA-IDS is to quantify distributional changes, identify stable features, and incorporate limited target-domain knowledge into the learning process in a controlled manner. In this respect, DEA-IDS provides a drift-aware and explainable architecture designed to improve operational reliability in cross-domain intrusion detection.

### 3.2. Explainability Layer

The Explainability Layer, which constitutes the first component of the DEA-IDS architecture, is based on SHAP (SHapley Additive exPlanations) analysis to investigate which features influence the model’s predictions. The primary objective of this layer is to make the contribution of individual features to the classification process explicit in both the source and target domains and to interpret changes in feature behavior during domain transfer. For this purpose, SHAP values were computed using the TreeExplainer interface. To ensure that observed differences between domains reflect domain shift rather than differences between separately trained models, SHAP values were computed on the same source-trained XGBoost model (the Baseline configuration defined in Section 4.6, trained once on CICIoT2023 using all 44 common features) applied consistently to both domains, rather than on aseparately trained Random Forest reference model as in the original submission. The analyses were performed separately on the CICIoT2023 source-domain training subsample and the CICIoMT2024 target-domain training subsample (the test split was not used for this analysis). By comparing the mean absolute SHAP values calculated for each feature, we examined which variables maintained similar importance across different network environments and which exhibited changes in their influence on the model’s predictions. The rank agreement between the two domains was further quantified using the Spearman rank correlation between the source and target mean-absolute-SHAP feature rankings, which was ρ=0.997 (p<0.001), indicating that the two domains rely on an almost identical feature-importance ordering under the same trained model. The SHAP analysis and the computation of the mean absolute SHAP values were performed automatically by the analysis pipeline developed in this study. Within the DEA-IDS architecture, SHAP analysis is not used directly for feature selection. Instead, it serves as a complementary mechanism for interpreting the results of the drift analysis. In particular, examining whether highly important features exhibit similar behavior across domains provides additional insight into the observed drift patterns. Therefore, the Explainability Layer is designed not as an optimization component that guides feature selection, but as a complementary analytical module that explains model behavior and supports the interpretation of drift analysis results. This makes it possible to examine not only the overall model performance but also how the features influencing the model’s predictions are affected by domain shift.

To make the role of each component unambiguous, we state explicitly: SHAP provides interpretability and post-hoc feature-importance analysis; the KS statistic (Section 3.3) performs stable-feature selection; and few-shot adaptation (Section 3.5) performs target-domain adaptation. These three mechanisms operate on the pipeline independently rather than through a single integrated optimization objective, and Section 5.16 shows empirically that a fourth, natural-seeming alternative—selecting features by SHAP importance rather than by drift stability—does *not* reproduce the FPR reduction achieved by KS-based stable feature selection, underscoring that “important” and “stable” are distinct properties of a feature and that only the latter is responsible for the reported operational improvement.

### 3.3. Drift Analysis Layer

The Drift Analysis Layer, which is the second component of the DEA-IDS architecture, was designed to compare the feature distributions between the source domain (CICIoT2023) and the target domain (CICIoMT2024). In this layer, two complementary statistical methods, the Kolmogorov–Smirnov (KS) test and the Wasserstein Distance, were employed to determine which features exhibit similar behavior across domains and which show significant distributional changes during domain transfer. The KS test evaluates the statistical similarity between two distributions by measuring the maximum difference between their cumulative distribution functions. The Wasserstein Distance, on the other hand, calculates the cost of transforming one distribution into another and provides additional information about the magnitude of the differences between the distributions. Using these two methods together makes it possible to examine not only whether differences exist between the distributions but also the extent of those differences. Both statistics were computed on the raw (unscaled) feature values, prior to the StandardScaler normalization described in Section 4.2. The KS statistic is scale-invariant, so this do es not affect the drift-based feature-selection criterion (KS < 0.1) used in this study. The Wasserstein distance, however, is scale-dependent, so its absolute magnitude (Table 1) should be interpreted relative to each feature’s native units and is not directly comparable across features with different scales (e.g., a Wasserstein distance of a few units for a proportion-like feature is not commensurate with a distance of thousands of units for a byte-count or rate feature); it is reported here as descriptive, complementary evidence rather than as a normalized measure of relative feature stability.

The drift analysis was performed only on benign (normal) network traffic samples. The primary reason for this is that false positives arise when normal network traffic is classified as an attack. Therefore, examining changes in the distribution of benign traffic directly contributes to evaluating the extent to which the model interprets legitimate user behavior differently in the target environment and to explaining the possible causes of increases in the False Positive Rate (FPR). In addition, this approach provides a more reliable statistical basis for the stable feature selection process performed in the subsequent stage. All 44 features in the common feature space were included in the drift analysis. The resulting measurements were used to identify features that exhibit more consistent behavior between the source and target domains and provided the statistical foundation for the stable feature selection process performed in the subsequent stage.

To avoid any use of target-domain test-set information during feature selection, drift statistics were computed exclusively between the CICIoT2023 training split and a disjoint pool of 100,000 samples drawn from the CICIoMT2024 *training* split; the CICIoMT2024 test set was not accessed at any point during drift analysis, threshold selection, or feature selection, and was used only for final model evaluation (Section 4.3). This protocol was verified in two independent ways. First, the Baseline configuration (Section 4.6), which does not depend on drift analysis or few-shot adaptation, reproduces the originally reported FPR on the untouched test set to within $10^{-4}$ (0.5469 vs. the previously reported 0.5468), confirming that no other aspect of the pipeline was altered. Second, the resulting leakage-free stable-feature set (Section 3.4) is identical to the one obtained when drift statistics were computed against the test split, indicating that, for this dataset pair, the reported improvements are not an artifact of test-set leakage.

### 3.4. Stable Feature Selection Layer

The Stable Feature Selection Layer aims to identify features that exhibit more consistent behavior between the source and target domains by utilizing the statistical results obtained from the drift analysis. The underlying assumption of this layer is that features exhibiting substantial distributional changes may negatively affect classifier decisions in cross-domain scenarios, whereas features that preserve their distributions can provide more reliable information across different network environments. During the feature selection process, the Kolmogorov–Smirnov (KS) statistics computed by the Drift Analysis Layer were used. The KS value obtained for each feature was evaluated, and variables with values below the predefined threshold were regarded as stable features. In this study, the criterion KS < 0.1 was adopted, and the filtering process was performed according to this threshold. The stable feature selection process was based entirely on the statistical drift measurements obtained from the Drift Analysis Layer. As a result of the filtering process, 21 out of the 44 features in the common feature space were selected as stable features. The selected features represent different aspects of network behavior, including protocol-related characteristics, TCP flag information, and flow statistics. Consequently, the resulting feature subset is not limited to a particular feature category but instead provides a balanced representation of different network characteristics. The threshold KS < 0.1 was selected to achieve a balance between eliminating features with substantial drift and preserving sufficient feature diversity. Applying this threshold retained 21 of the 44 features in the common feature space. To verify that this specific cutoff is not arbitrary, we additionally swept the threshold over {0.05,0.075,0.10,0.125,0.15,0.20}, retaining between 17 and 22 features (Table 2, Section 5.8); the resulting FewShot_Stable configuration maintained an FPR between 0.0004 and 0.0008 across this entire range, indicating that the reported false-positive-rate reduction is not sensitive to the exact choice of threshold.

The resulting stable feature subset constitutes the feature space used in the subsequent stages of the DEA-IDS architecture. The objective of this approach is to reduce the influence of features that exhibit significant distributional changes in the target domain and to perform model learning using more consistent features. The list of stable features was kept unchanged throughout all experiments, thereby ensuring the reproducibility of the reported results. This layer represents one of the core components of the DEA-IDS architecture by directly transferring the statistical information obtained from the drift analysis into the modeling process. Consequently, the drift analysis functions not only as an explanatory tool for reporting differences between datasets but also as an active mechanism that guides the feature filtering process and directly influences the subsequent learning stage.

### 3.5. Few-Shot Adaptation Layer

The Few-Shot Adaptation Layer, which is the final component of the DEA-IDS architecture, was designed to enable the model trained on the source domain to better adapt to the conditions of the target domain. Rather than modifying the model architecture, this approach follows a data-driven adaptation strategy based on augmenting the training data. During the adaptation process, a limited number of labeled samples were selected from the CICIoMT2024 dataset representing the target domain and combined with the source-domain training data. According to the experimental protocol adopted in this study, 1000 labeled samples were obtained from the target domain and added to the 100,000-sample source training set derived from the CICIoT2023 dataset. As a result, an augmented training set containing information from both the source and target domains was constructed. The adaptation set consisting of 1000 samples was selected to represent realistic operational scenarios in which only a limited amount of labeled data is available from the target domain. This sample size corresponds to approximately 1% of the source training set and allows target-domain information to be incorporated into the model while preserving the source domain as the dominant component of the training data. To assess the sensitivity of this choice, the adaptationsize was swept over {100,500,1000,2000} samples, each repeated across five independently sampled adaptation sets (seeds 42, 101, 202, 303, 404), and results are reported as mean ± standard deviation (Table 3, Section 5.9). Following reviewer requests for a wider size grid and more seeds, this sweep was subsequently extended to {10,50,100,250,500,1000,2000} samples × 10 seeds (70 conditions per configuration; Table 3). When combined with stable feature selection, the resulting FPR was identical (0.0004) across all seven sample sizes and all ten seeds (standard deviation =0 at every size, including at just 10 few-shot samples, where the sampled adaptation set contained on average only 0.1 benign target-domain flows), whereas without stable feature selection FPR varied substantially with both sample size and seed (e.g., mean FPR =0.540±0.020 at 10 samples, decreasing to 0.106±0.025 at 2000 samples, with a minimum-to-maximum range as wide as [0.056,0.135] at the 2000-sample size alone), indicating that stable feature selection is the primary source of the method’s robustness to the few-shot sampling procedure.

The underlying assumption of this approach is that even a small set of samples representing the target domain can provide the model with additional information about the data distribution of the new environment. In this way, the model is able to incorporate traffic patterns specific to the target domain into the learning process instead of relying solely on patterns learned from the source domain. The few-shot adaptation mechanism was designed to operate together with the Stable Feature Selection Layer. Consequently, the model benefits from the limited information obtained from the target domain while learning from features identified as being more robust to distributional changes. This structure aims to mitigate the effects of domain shift by combining statistically selected stable features with limited target-domain adaptation. To ensure the reproducibility of the experiments, the sampling procedure was performed using a fixed random seed (42), and the number of samples selected from the target domain was kept constant throughout all experiments.

### 3.6. Evaluation Strategy

The proposed DEA-IDS framework was evaluated under a cross-domain intrusion detection scenario in which the model was trained on the CICIoT2023 dataset and tested on the CICIoMT2024 dataset. This experimental setting was designed to assess the robustness of DEA-IDS under distributional differences between the source and target domains. To investigate the contribution of each component of DEA-IDS, an ablation study consisting of four different experimental configurations was conducted. The first configuration (Baseline) uses all 44 features in the common feature space and is trained solely on the source-domain data. The secondconfiguration (Stable-only) restricts training to the 21 stable features identified through drift analysis but, like Baseline, does not use any target-domain adaptation samples; this configuration was added to isolate the individual contribution of stable feature selection from its interaction with few-shot adaptation. The third configuration (FewShot) preserves all common features while augmenting the source-domain training data with 1000 labeled samples selected from the target domain. The final configuration (FewShot + Stable) combines the few-shot adaptation approach with the 21 stable features identified through the proposed drift analysis and performs model training on the resulting reduced feature space. Model performance was evaluated using Accuracy, Precision, Recall, F1-Score, ROC-AUC, False Positive Rate (FPR), and False Negative Rate (FNR). Although conventional classification metrics were reported to provide an overall performance assessment, particular emphasis was placed on the False Positive Rate (FPR) because reducing false alarms is a primary objective of the proposed framework. High false positive rates may increase analyst workload, contribute to alarm fatigue, and reduce the operational usability of intrusion detection systems in real IoT–IoMT environments. Therefore, FPR was considered one of the primary evaluation metrics for assessing cross-domain performance. The same preprocessing procedure was applied in all experiments, and a fixed random seed (SEED = 42) was used to ensure the reproducibility of the results. The training split of the CICIoT2023 dataset was used as the source-domain training data, while all evaluations were performed on the CICIoMT2024 test set under the same experimental protocol. The effectiveness of the proposed DEA-IDS framework was first evaluated through the ablation study and subsequently compared with a Multi-Layer Perceptron (MLP)-based intrusion detection model that is widely used in the literature.

### 3.7. Algorithm Summary

To improve reproducibility, the end-to-end DEA-IDS procedure described in Section 3.1, Section 3.2, Section 3.3, Section 3.4, Section 3.5 and Section 3.6 is summarized below as a concise, sequential algorithm.

1. Source data preparation. Load the CICIoT2023 source dataset; restrict to the 44 common features (Section 4.1); impute missing values with 0; binarize labels (Benign =0, all attack categories =1); draw a fixed-seed random sample of up to 100,000 rows.
2. Target adaptation-data selection. Independently load the CICIoMT2024 *training* split (never the test split at this stage); apply the same feature restriction, imputation, and binarization; draw a fixed-seed random sample of up to 100,000 rows to form the target training pool; draw a fixed-seed sub-sample of 1000 rows from this pool as the few-shot adaptation set (Section 3.5).
3. Drift calculation. Restrict both the source sample and the target training pool to benign-labeled rows; for each of the 44 common features, compute the Kolmogorov–Smirnov statistic and the Wasserstein distance between the source-benign and target-benign distributions (Section 3.3).
4. Stable-feature selection. Retain the subset of features with KS <0.1 (21 of 44 features; Section 3.4).
5. Few-shot augmentation. Concatenate the source training sample (Step 1) with the 1000-sample target adaptation set (Step 2) to form the augmented training set.
6. Scaling and XGBoost training. Fit a StandardScaler on the training data (source-only for the Baseline/Stable-only configurations, or the augmented set for the FewShot/FewShot_Stable configurations), restricted to either all 44 features or the 21 stable features depending on the configuration (Section 4.6); train an XGBoost classifier (hyperparameters in Section 4.7) on the scaled training data.
7. Final target-test evaluation. Apply the (source-domain-fitted) scaler to the CICIoMT2024 *test* split—accessed here for the first and only time—and evaluate the trained classifier, reporting Accuracy, Precision, Recall, F1-score, ROC-AUC, FPR, FNR, and the extended metrics in Section 5.14.

Steps 1–6 are repeated independently for each of the four ablation configurations (Baseline, Stable-only, FewShot, FewShot_Stable; Section 4.6) and, where applicable, for each KS threshold (Section 5.8) or few-shot sample-size/seed combination (Section 5.9) under evaluation; Step 7 is always performed against the same, untouched CICIoMT2024 test split.

## 4. Experimental Setup

### 4.1. Datasets

Two publicly available network traffic datasets, CICIoT2023 and CICIoMT2024, were selected to represent the source and target domains, respectively. Both datasets were developed by the Canadian Institute for Cybersecurity (CIC) and consist of network flow records labeled as either benign or malicious traffic. The CICIoT2023 dataset [35] was used as the source domain. This dataset contains network traffic collected from various IoT devices and includes different attack categories, such as Distributed Denial-of-Service (DDoS), Denial-of-Service (DoS), Mirai botnet, and various scanning attacks. Model training was performed exclusively on the training split of the CICIoT2023 dataset. The CICIoMT2024 dataset [36] was used as the target domain. This dataset represents realistic Internet of Medical Things (IoMT) network environments and was employed as the test dataset for cross-domain evaluation. Following the experimental protocol, model performance was evaluated on the CICIoMT2024 test set. As described in Section 3.5, the few-shot adaptation stage incorporated 1000 randomly selected labeled samples from the CICIoMT2024 training set into the augmented training data. To establish a common feature space between the source and target domains, 44 network flow features shared by both datasets were selected. These features include TCP flag counters (e.g., FIN, SYN, and RST), protocol indicators (e.g., HTTP, DNS, ARP, ICMP, and SSH), and flow statistics (e.g., IAT, Variance, Weight, and Number). Consequently, both datasets were represented within the same feature space, enabling direct cross-domain evaluation. The labels in both datasets were converted into a binary classification problem, where Benign samples were assigned the label 0, while all attack categories were merged under the Attack (1) label. To control the computational cost while preserving data diversity, a maximum of 100,000 samples was used from each dataset in all experiments. Sampling was simple random sampling without replacement (pandas.DataFrame.sample with a fixed random seed), applied independently to each dataset; it was not stratified by class or by attack family, so the resulting class distribution reflects each dataset’s native class imbalance. The exact benign/attack counts for every data split used in this study are as follows: source-domain training pool (CICIoT2023), 2377 benign/97,623 attack; target-domain training pool (CICIoMT2024), 2745 benign/97,255 attack; target-domain test set (CICIoMT2024), 2368 benign/97,632 attack; and the 1000-sample few-shot adaptation subset drawn from the target training pool, 27 benign/973 attack. The same sampling strategy was maintained across all experimental configurations to ensure the comparability and reproducibility of the reported results.

### 4.2. Data Preprocessing

The data preprocessing procedure was applied consistently across all experimental configurations and was designed to ensure the comparability of the source and target domain data. In the first stage, the original feature space of both datasets was reduced to the 44 common features identified previously. Consequently, the CICIoT2023 and CICIoMT2024 datasets were represented within the same feature space, providing a common basis for cross-domain analysis. In the second stage, missing values in the datasets were addressed. Missing observations (NaN) were replaced with 0 using zero imputation to preserve all samples throughout the analysis. This prevented the removal of any observations from the datasets while maintaining the integrity of the feature space. Next, the multiclass label structure in both datasets was converted into a binary classification problem. Benign samples were assigned the label 0, whereas all attack categories were merged under the Attack (1) label. This transformation established a common classification framework for both the source and target domain datasets. After the label transformation, feature values were normalized using the StandardScaler to reduce the effect of differences in feature scales on model performance. To correct an inconsistency identified during the second round of review, we state precisely: the scaler is fitted on whatever data a given configuration is trained on—source-domain training data only for the Baseline and Stable-only configurations, and the augmented (source + 1000-sample few-shot) training set for the FewShot and FewShot_Stable configurations (consistent with the Algorithm Summary, Section 3.7)—and is then applied, unchanged, to the held-out CICIoMT2024 test set for evaluation. This strategy prevented information from the target *test* split from influencing the normalization process and ensured that the cross-domain evaluation remained free from test-set data leakage; scaling has limited effect on XGBoost’s tree-based decision structure in any case, so this reconciliation does not change any previously reported number. Finally, to ensure the reproducibility of the experiments, SEED = 42 was used for all sampling and random selection procedures. Finally, all sampling and random selection procedures were performed using a fixed random seed (SEED = 42), ensuring consistent data partitioning across all experimental configurations.

### 4.3. Cross-Domain Experimental Design

The experimental protocol of this study was designed as a cross-domain evaluation framework to assess the generalization capability of intrusion detection systems across different network environments. Within this framework, the CICIoT2023 dataset was used as the source domain, while the CICIoMT2024 dataset served as the target domain.

Source Domain: CICIoT2023 [35]—training data
Target Domain: CICIoMT2024 [36]—test data

Following the experimental protocol, the model was trained exclusively on the source-domain data and subsequently evaluated on the target-domain test set. This design makes it possible to examine the extent to which the decision boundaries learned from the source domain can generalize to a target domain with different traffic characteristics. Consequently, the impact of distributional differences between network environments on classification performance can be directly observed. Preventing data leakage was adopted as a fundamental principle throughout the experimental design. Accordingly, the target-domain test set was not used at any stage of the training process or during the fitting of the feature scaler. The 1000 labeled target-domain samples employed during the few-shot adaptation stage were selected exclusively from the training split of the CICIoMT2024 dataset, while the test data remained completely unseen throughout the adaptation process. The same training and test partitions were maintained across all experimental configurations. As a result, the Baseline, FewShot, and FewShot_Stable configurations were evaluated on the identical target-domain test set, ensuring that any observed performance differences were attributable to the proposed adaptation and feature selection strategies rather than to differences in data partitioning.

### 4.4. Evaluation Metrics

The performance of the proposed DEA-IDS architecture was assessed using Accuracy, Precision, Recall, F1-score, ROC-AUC, and False Positive Rate (FPR). Together, these metrics provide a comprehensive assessment of binary classification performance by capturing both overall predictive capability and the ability to detect malicious traffic while minimizing false alarms.

The mathematical definitions of the evaluation metrics are given below.

(1) $$\mathrm{Accuracy}=\frac{\mathrm{TP}+\mathrm{TN}}{\mathrm{TP}+\mathrm{TN}+\mathrm{FP}+\mathrm{FN}}$$

(2) $$\mathrm{Precision}=\frac{\mathrm{TP}}{\mathrm{TP}+\mathrm{FP}}$$

(3) $$\mathrm{Recall}=\frac{\mathrm{TP}}{\mathrm{TP}+\mathrm{FN}}$$

(4) $$F_{1}=\frac{2PR}{P+R}$$

(5) $$\mathrm{FPR}=\frac{\mathrm{FP}}{\mathrm{FP}+\mathrm{TN}}$$

In these equations, TP, TN, FP, and FN correspond to the numbers of true positives, true negatives, false positives, and false negatives, respectively. The variables *P* and *R* denote Precision and Recall.

ROC-AUC (Area Under the Receiver Operating Characteristic Curve) was also reported because it evaluates how well the classifier distinguishes attack traffic from benign traffic without relying on a specific decision threshold.

In this study, the False Positive Rate (FPR) was considered the primary operational evaluation metric. High false positive rates may increase the workload of security analysts and raise the risk of overlooking genuine threats in security operation centers [37]. For this reason, although Accuracy, Precision, Recall, F1-score, and ROC-AUC are reported, greater attention is given to FPR because it more directly reflects the operational reliability of the proposed DEA-IDS framework.

### 4.5. Literature Baseline Benchmark

A Multilayer Perceptron (MLP)-based neural network was adopted as the baseline model for comparison with the proposed DEA-IDS architecture. Owing to its widespread use in intrusion detection research, the MLP is commonly employed as a reference model for evaluating newly proposed methods [4]. In this study, it represents a conventional supervised learning approach that does not incorporate any domain adaptation mechanism. The MLP model was configured with two hidden layers containing 64 and 32 neurons, respectively, and ReLU was used as the activation function. The model was implemented using the MLPClassifier provided by the scikit-learn library. A fixed random seed (SEED = 42) was used, the maximum number of training iterations was set to 200, and early stopping was enabled. The model was trained using 100,000 samples selected from the training split of the CICIoT2023 dataset. To ensure a fair comparison, the MLP and DEA-IDS models were evaluated using the same data partitioning strategy, the same common feature space, and the same preprocessing pipeline. This experimental design was intended to minimize the influence of data preparation procedures on the results and to ensure that both models were assessed under identical experimental conditions. The MLP baseline was evaluated under two scenarios. In the first scenario, the model was tested on the IoT test set, which follows the same distribution as the training data, in order to establish its performance under independent and identically distributed (IID) conditions. In the second scenario, the same model was directly applied to the CICIoMT2024 test set without any domain adaptation. This enabled an assessment of how a conventional neural network trained on an IoT environment performs in an IoMT target domain when deployed in an IoMT environment with distributional differences.

### 4.6. Ablation Study Design

The contribution of each component of the proposed DEA-IDS framework was investigated through an ablation study. Four experimental configurations were evaluated, with each configuration differing from the others by only one component.

Configuration 1—Baseline: An XGBoost classifier was trained on the CICIoT2023 training dataset using the complete set of common features. This configuration served as the source-domain baseline without applying domain adaptation or feature selection.

Configuration 2—Stable-only: An XGBoost classifier was trained on the CICIoT2023 training dataset (no target-domain adaptation samples) using only the 21 stable features identified through drift analysis. This configuration isolates the effect of stable feature selection in the absence of few-shot adaptation.

Configuration 3—FewShot: An XGBoost classifier was trained on an augmented training set created by adding 1000 randomly selected labeled samples from the CICIoMT2024 training split to the CICIoT2023 training data. All common features were retained so that the effect of few-shot adaptation could be evaluated independently.

Configuration 4—FewShot_Stable: An XGBoost classifier was trained on the same augmented training set using only the 21 stable features identified through the proposed drift analysis. This configuration represents the complete DEA-IDS framework, where few-shot adaptation and stable feature selection are applied together.

All four configurations were evaluated on the same CICIoMT2024 test set using an identical preprocessing pipeline and the same evaluation metrics. By keeping the experimental protocol unchanged, the individual contributions of few-shot adaptation and stable feature selection, as well as their combined effect on the overall performance, could be examined directly. All four configurations used identical XGBoost hyperparameters (n_estimators = 200, max_depth = 6, learning_rate = 0.1, subsample = 0.8, colsample_bytree = 0.8, scale_pos_weight set automatically from the training-set class ratio, random_state = 42); the complete hyperparameter, software-version, and per-phase runtime log is provided in Section 4.7 for reproducibility.

### 4.7. Reproducibility

All experiments in this revision were run with Python 3.12.2, numpy 2.4.4, pandas 3.0.2, scikit-learn 1.8.0, xgboost 3.2.0, scipy 1.17.1, and shap 0.51.0, on an 8-core machine (macOS, arm64). The complete leakage-free ablation (four configurations × two target domains), the KS-threshold sensitivity sweep (six thresholds), the few-shot sample-size×seed sweep (four sizes × five seeds × two configurations, 40 model fits), the feature-selection control comparison (three additional feature sets), and the SHAP re-analysis together completed in 233 s of model-fitting and analysis time; the dominant cost was loading the raw CSV source files (source-domain training data: 21.8 s; target-domain training pool: 19.8 s; target-domain test set: 4.0 s; CICAPT-IIoT2024 secondary target domain: 96.4 s). All configurations share the XGBoost hyperparameters listed in this section, with scale_pos_weight set automatically to the benign/attack ratio of the corresponding training set (approximately 0.0243 for the source-only configurations and 0.0244 for the few-shot-augmented configurations, reflecting the 1000 added target-domain samples). No hyperparameter tuning or model-selection search (e.g., grid search, cross-validation-based selection) was performed for any classifier reported in this study: the XGBoost hyperparameters (this section), the Random Forest hyperparameters (Section 5.18), and the Logistic Regression configuration (Section 5.19) were each fixed a priori to conventional default-adjacent values and applied identically across all experimental configurations within a given classifier family, so that observed performance differences reflect the feature-selection and adaptation strategies under study rather than differential tuning effort. A fixed random seed (42) was used for all data sampling and model training in the main results; Section 5.9 additionally reports results across five independent seeds to characterize sampling variability. Peak resident memory usage, measured for the additional gap-closure analyses in this revision (Section 5.14, Section 5.15, Section 5.16 and Section 5.17: extended-metric computation, the Wasserstein-alone/combined/ANOVA/RFE/PCA feature-selection comparisons, and the Random Forest baseline, run as a single process holding the 100,000-row source and target training samples in memory simultaneously) was approximately 1.5 GB; the earlier leakage-free ablation and sensitivity analyses (loading up to four datasets of up to 100,000 rows each) exhibited comparable or lower peak memory usage. At inference time, scoring the 100,000-row CICIoMT2024 test set with the final trained XGBoost FewShot_Stable model (21 features) completed as part of the sub-second predict_proba calls already included in the per-configuration timings above, and does not materially add to the reported runtimes. Code availability: the complete analysis pipeline used to produce this revision (leakage-free drift analysis, all ablation and sensitivity experiments, SHAP re-analysis, attack-family breakdown, and the additional baselines and statistical tests reported in Section 5.8, Section 5.9, Section 5.10, Section 5.11, Section 5.12, Section 5.13, Section 5.14, Section 5.15, Section 5.16 and Section 5.17) is implemented as a set of self-contained Python scripts and is available from the corresponding author upon reasonable request.

## 5. Results

### 5.1. Cross-Domain Performance Evaluation

The cross-domain performance of the proposed DEA-IDS framework was evaluated by training the models on the CICIoT2023 source-domain dataset and testing them on the CICIoMT2024 target-domain test set. To quantify the impact of the proposed adaptation strategy, the obtained results were compared with those of a baseline model trained only on the source-domain data without any domain adaptation.

The results below were re-derived under a leakage-free protocol (Section 3.3) in which drift statistics and few-shot adaptation samples are drawn exclusively from the CICIoMT2024 training split, and a fourth configuration (Stable-only) was added to isolate the individual contribution of stable feature selection (Section 4.6). The experimental results are presented in Table 4. Although all four configurations achieved high classification performance, noticeable differences were observed in their operational characteristics, particularly with respect to the False Positive Rate (FPR). The baseline model produced an FPR of 0.5468 (reproduced here as 0.5469 under the leakage-free protocol), whereas the complete DEA-IDS framework (FewShot_Stable) reduced this value to 0.0004 (1 false positive out of 2368 benign test flows), corresponding to an approximately 99.9% reduction in false alarms. Notably, the newly added Stable-only configuration—which uses no target-domain adaptation samples at all—already achieves the same FPR (0.0004) as the full FewShot_Stable configuration. This indicates that, in this leakage-free re-analysis, the false-positive-rate reduction is attributable almost entirely to drift-aware stable feature selection rather than to few-shot adaptation; the incremental contribution of few-shot adaptation manifests instead as a reduction in false negatives (1176 for Stable-only vs. 1077 for FewShot_Stable; see Table 5) and a corresponding increase in F1-score, rather than a further reduction in FPR. Incorporating few-shot adaptation alone (without stable feature selection) already improved cross-domain performancerelative to the Baseline by reducing the effect of domain shift, but produced a markedly higher FPR (0.1660) than either stable-feature configuration. Consistent with a request in review to avoid overclaiming causality, we phrase this observation cautiously: these results are associated with, and consistent with the interpretation that, selecting stable features before adaptation is an effective strategy for enhancing robustness across domains while preserving high intrusion detection performance, under the experimental conditions tested in this study (Section 4); we do not claim this association has been established as a universal causal relationship that would necessarily hold under different datasets, feature sets, or classifiers, and Section 5.16 and Section 5.17 (respectively) show that the effect is specific to drift-based selection rather than to feature selection in general, and is not identical in magnitude across classifiers.

Table 5 reports the underlying raw confusion-matrix counts on the CICIoMT2024 test set (2368 benign and 97,632 attack flows) for all four configurations, addressing the reviewers’ request for absolute error counts rather than normalized rates alone.

### 5.2. Benchmark Results

A Multilayer Perceptron (MLP)-based neural network was used as the benchmark model to provide a reference for evaluating the proposed DEA-IDS framework. Because the MLP is one of the most commonly used baseline models in intrusion detection research, it was selected to represent a conventional supervised learning approach. Rather than introducing a new state-of-the-art benchmark, this comparison aims to illustrate how domain shift affects the operational behavior of a standard IDS model and to provide a meaningful reference for assessing the proposed DEA-IDS framework. For this reason, the MLP was evaluated under both independent and identically distributed (IID) conditions (IoT → IoT) and a cross-domain setting (IoT → IoMT).

Table 6 summarizes the benchmark results. Under IID conditions, the MLP achieved consistently high scores across all evaluation metrics, showing that the model performed well when the training and test data followed the same distribution. Its behavior changed after deployment in the IoMT target domain. Although the Accuracy and F1-score remained largely unchanged, the operational metrics deteriorated noticeably. The ROC-AUC decreased from 0.9964 to 0.9443, indicating a reduced ability to distinguish malicious traffic from benign traffic under domain shift. At the same time, the False Positive Rate (FPR) increased from 0.1430 to 0.3255, representing an approximately 2.3-fold increase in false alarms.

These observations suggest that domain shift can have only a limited effect on conventional classification metrics while substantially reducing operational reliability. Despite maintaining a high F1-score in both evaluation scenarios, the marked increase in FPR indicates that considerably more benign traffic was incorrectly identified as malicious in the target domain. This result also shows that relying only on conventional performance metrics may conceal the practical impact of domain shift. Therefore, operational measures, particularly the False Positive Rate (FPR), should be considered an essential part of evaluating cross-domain intrusion detection systems.

### 5.3. Comparison with Literature Benchmarks

To provide additional context for the performance of DEA-IDS, this section compares its cross-domain results with those reported by directly related cross-domain and domain-adaptation studies. Mahbub et al. [14] proposed a feature-based domain adaptation method (Classwise Wasserstein Distance combined with Particle Swarm Optimization and Logistic Regression), using the ACIIoT2023 dataset as the source domain and CICIoMT2024 as the target domain, achieving an F1-score of 94.23% overall; the corresponding False Positive Rate was not reported. Elangovan et al. [23] evaluated multiple classifiers (Random Forest, Gradient Boosting, MLP, Autoencoder, and a lightweight 1D-CNN) across NIDS benchmark datasets spanning 2017–2024 and reported that, under cross-domain transfer, Macro-F1 decreased to 0.69–0.78 while benign false-positive rates increased to as much as 0.30. Liu et al. [24] proposed a multi-constraint transfer approach with auxiliary domains for IoT intrusion detection under unbalanced sample distributions, reporting an average accuracy of 96.4% across four datasets, without reporting FPR.

As summarized in Table 7, DEA-IDS achieves a higher F1-score (0.9944) than each of these directly related cross-domain studies, while additionally reporting an FPR of 0.0004—a metric that only Elangovan et al. [23] also provide, and for which DEA-IDS shows a substantially lower value (0.0004 vs. up to 0.30). Where a direct point of comparison exists on a metric other than F1, DEA-IDS is also higher: its ROC-AUC (0.9984) exceeds the 0.956 best-case AUC reported by Vu et al. [25] and its accuracy (0.9892) exceeds the 0.964 average accuracy reported by Liu et al. [24], although, as with the F1 comparison above, these studies evaluate different source–target dataset pairs and are not a fully controlled comparison.

Independently, Doménech et al. [38] evaluated intrusion detection models trained on CICIoT2023 and tested on CICIoMT2024—the identical source-target dataset pair used in this study—and reported that, without domain-specific adaptation, the F1-score degraded by up to 66.87%. This finding, obtained using the same dataset pair as the present work, is consistent with the substantial FPR degradation observed for the Baseline configuration in Table 4 (FPR = 0.5468) and further supports the conclusion that direct transfer from IoT to IoMT network traffic is unreliable without an explicit adaptation strategy. Additionally, Vu et al. [25] proposed an autoencoder-based deep transfer learning model (M2DA) evaluated across nine cross-device IoT datasets, reporting AUC scores of up to 0.956 in the best-performing source-target configuration; this study did not report point F1-score or FPR values, relying instead on ROC-based evaluation.

These comparisons should be interpreted with some caution: differences in source datasets, feature sets, attack taxonomies, and preprocessing pipelines across studies limit strict one-to-one comparability, and FPR is rarely reported in the reviewed literature. Nevertheless, the available evidence indicates that DEA-IDS performs competitively with, and reports a broader set of operational metrics than, existing cross-domain intrusion detection approaches.

For additional context, Table 8 summarizes accuracy figures reported by several recent studies that trained and evaluated their models directly on the CICIoMT2024 dataset, without any cross-domain transfer. Shebl et al. [39] reported accuracies of 99.98% and 99.86% for binary and multiclass classification, respectively. Benahmed et al. [40] achieved 98.81% accuracy across 19 attack types using a hybrid BiLSTM-DNN architecture. Shaikh et al. [41] reported 99.58% accuracy for binary classification and 77.73% for an 18-class multiclass setting. Sharma and Shambharkar [42] reported 99.49%, 99.12%, and 98.56% accuracy for binary, multiclass, and extended classification tasks, respectively. As requested in review, these figures are presented as a contextual reference point rather than as a strict experimental upper bound: they come from different studies, models, and (in some cases) evaluation protocols, and are reported here only to situate DEA-IDS’s cross-domain performance relative to what has been achieved without the additional difficulty of domain shift. They should not be read as a directly comparable ceiling, since the corresponding models are trained and tested on data drawn from the same distribution, without the additional difficulty introduced by domain shift. Notably, the F1-score achieved by DEA-IDS under the more challenging IoT-to-IoMT cross-domain scenario (0.9944) approaches this in-distribution ceiling, indicating that the proposed framework substantially closes the gap typically introduced by domain shift.

### 5.4. Ablation Results

An ablation study was conducted to quantify the contribution of each component of the proposed DEA-IDS framework under the cross-domain IoT-to-IoMT evaluation scenario. The results are summarized in Table 9. The Baseline configuration, trained exclusively on the source-domain data using all 44 common features, achieved high overall classification performance. However, it produced a False Positive Rate (FPR) of 0.5468, indicating that the model incorrectly classified a substantial proportion of benign IoMT traffic as malicious after deployment in the target domain. This observation confirms that conventional training on the source domain alone is insufficient to ensure reliable cross-domain operation. The newly added Stable-only configuration—which restricts training to the 21 drift-stable features but uses no target-domain adaptation samples at all—already reduced the FPR from 0.5469 to 0.0004 (1 false positive out of 2368 benign test flows; Table 5), while F1-score improved from 0.9926 to 0.9939. This is the single most important refinement of this revision: because Stable-only alone reaches the same FPR as the full FewShot_Stable configuration, drift-aware stable feature selection—not few-shot adaptation—is the primary driver of the reported false-positive-rate reduction.

Introducing FewShot adaptation by incorporating 1000 labeled target-domain samples without stable feature selection improved the model’s ability to adapt to the target environment relative to Baseline. Accuracy changed from 0.9855 to 0.9941, while the F1-score improved from 0.9926 to 0.9970. The FPR decreased from 0.5469 to 0.1660—a real but comparatively modest reduction next to the Stable-only result above, and still roughly 400 times higher than either stable-feature configuration. These results demonstrate that even a limited amount of labeled target-domain data can alleviate part of the impact of domain shift, but that feature-level drift, rather than the absence of target-domain samples, is the dominant cause of the Baseline’s elevated FPR.

The complete DEA-IDS framework (FewShot_Stable), which combines few-shot adaptation with stable feature selection, matched the Stable-only configuration’s FPR of 0.0004 while further improving F1-score to 0.9944 and reducing false negatives from 1176 to 1077 (Table 5) relative to Stable-only. In addition, the ROC-AUC increased to 0.9984, indicating improved discrimination between benign and malicious traffic under cross-domain conditions.

Overall, the ablation study demonstrates that the two proposed components provide distinct rather than symmetric benefits: drift-aware stable feature selection is responsible for essentially the entire false-positive-rate reduction, whereas few-shot adaptation’s incremental role—on top of stable feature selection—is to improve recall and reduce false negatives rather than to further suppress false alarms. This refines, without overturning, the original conclusion: combining both components still produces the most robust overall operational performance (lowest FPR together with the highest F1-score and ROC-AUC among all four configurations), and reducing feature instability prior to adaptation remains essential for reliable cross-domain intrusion detection—but the mechanism by which each component contributes is now precisely attributed.

### 5.5. SHAP Analysis

**Figure 2:**
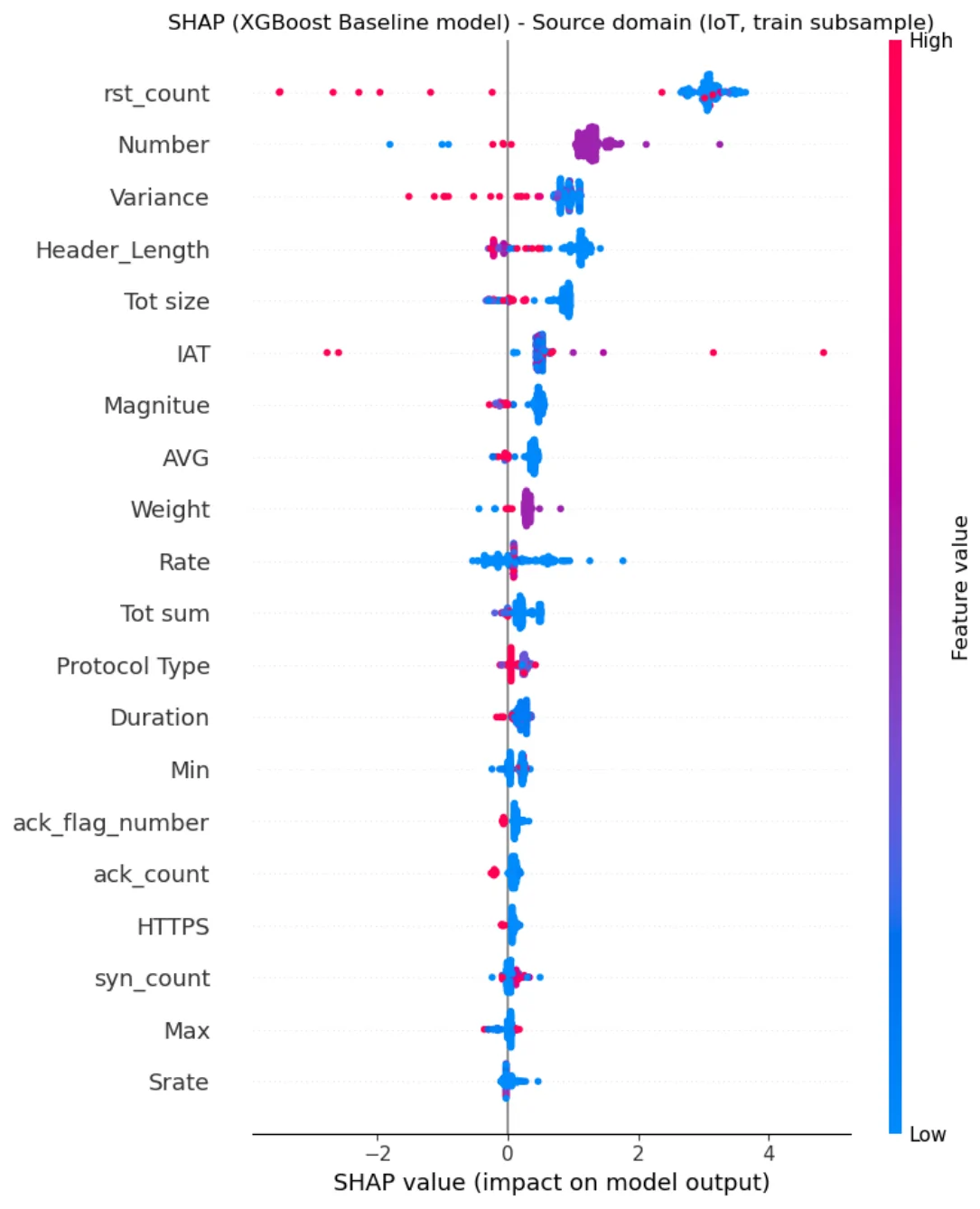
SHAP summary plot obtained from the source-domain (CICIoT2023) evaluation (revised: computed on the source-trained XGBoost Baseline model rather than a separate Random Forest reference model, evaluated on a held-out training subsample).

**Figure 3:**
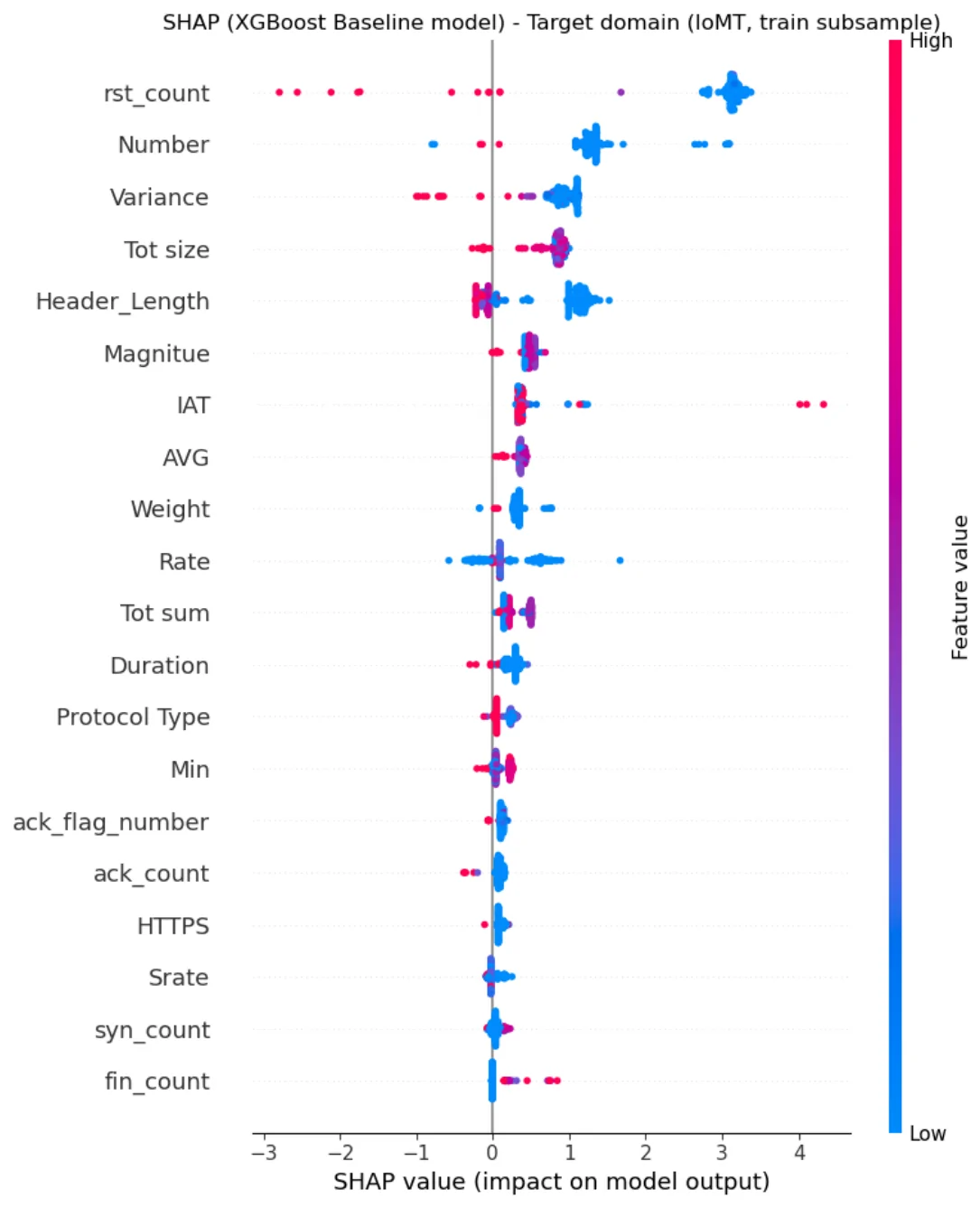
SHAP summary plot obtained from the target-domain (CICIoMT2024) evaluation (revised: same XGBoost model as Figure 2, evaluated on a held-out training subsample; test data were not used).

To investigate how feature contributions change across domains, a SHAP-based explainability analysis was performed using the source-domain (CICIoT2023) and target-domain (CICIoMT2024) datasets, computed on the same source-trained XGBoost Baseline model applied to held-out training subsamples from both domains (Section 3.2). The resulting SHAP summary plots are presented in Figure 2 and Figure 3 respectively. As shown in Figure 2, the most influential features in the source domain include rst_count, Number, Variance, Header_Length, and Tot size. A similar pattern can be observed in Figure 3, where these features remain among the highest-ranked contributors in the target domain (with only a minor rank swap between Header_Length and Tot size). This consistency indicates that the classifier preserves its overall decision mechanism despite the transition from IoT to IoMT environments, and is confirmed quantitatively by a Spearman rank correlation of ρ=0.997 (p<0.001) between the full source and target feature-importance rankings. Nevertheless, noticeable changes in the relative importance of several features can also be observed. In particular, the IAT feature becomes more influential in the target domain, while modest ranking changes are also visible for several traffic statistics and protocol-related features. These observations suggest that although the overall importance structure remains largely stable, the contribution of individual features is affected by the distributional differences between the two domains. Overall, the SHAP analysis reveals the coexistence of both stable and domain-sensitive features. The preservation of several highly ranked features indicates that a subset of network characteristics remains consistently informative across domains, whereas the observed changes in feature importance suggest that domain shift also influences the model’s decision process. These findings are consistent with the statistical drift analysis presented in the following section and provide qualitative support for the proposed drift-aware stable feature selection strategy.

### 5.6. Drift Analysis

To quantify the distributional differences between the source domain (CICIoT2023) and the target domain (CICIoMT2024), the Kolmogorov–Smirnov (KS) statistic and Wasserstein distance were computed for all common features. The results indicate that while several features exhibit substantial distributional changes after domain transfer, others maintain highly similar distributions across the two datasets. As in Section 3.3, the figures below are computed under the leakage-free protocol (CICIoT2023 training split vs. a disjoint CICIoMT2024 training pool, benign traffic only); the CICIoMT2024 test split was not used. The features with the largest distribution shifts are summarized in Table 1. In particular, psh_flag_number, Srate, Rate, HTTPS, and Duration produced the highest KS statistics, indicating pronounced statistical differences between the source and target domains. Similarly, the large Wasserstein distances observed for Rate and Srate demonstrate that these features differ not only statistically but also in the magnitude of their distributions. These findings suggest that these variables are highly sensitive to domain shift and therefore may negatively influence cross-domain intrusion detection performance if directly transferred without adaptation. In contrast, several features exhibit relatively stable behavior across both domains. As shown in Table 10, Drate, ece_flag_number, rst_flag_number, fin_flag_number, and HTTP all produced KS values below 0.1, together with very small Wasserstein distances. These results indicate that their underlying distributions remain largely unchanged despite the transition from IoT to IoMT environments, making them more suitable candidates for cross-domain learning. The complete list of all 44 features with their KS statistics, KS *p*-values, and Wasserstein distances were computed as part of this leakage-free analysis, and the 21 stable features together with their exact drift measurements are additionally reproduced in full in Table 11.

A methodological clarification is warranted regarding the use of the KS statistic as a selection criterion. The KS *statistic* (the maximum distance between the two empirical cumulative distribution functions, bounded in [0,1]) is used here as a continuous, scale-invariant measure of distributional distance—an effect-size-like criterion—rather than as a hypothesis test conducted at a fixed significance level. This choice was deliberate: with the large sample sizes used in this study (thousands of benign flows per domain), the KS *p-value* is effectively 0 (or numerically indistinguishable from 0) for the majority of the 44 features (Table 1 reports *p*-values as low as $10^{-323}$), so a conventional significance-threshold decision rule (e.g., p<0.05, with or without a Bonferroni or Benjamini–Hochberg correction for the 44 simultaneous tests) would classify nearly every feature as exhibiting statistically significant drift, including features with a negligible KS statistic (e.g., rst_flag_number, KS =0.0066, p≈1; see Table 11) whose CDFs are nonetheless nearly identical in practice. We therefore treat the KS statistic’s magnitude, not its associated *p*-value, as the operative criterion for feature stability, consistent with its common use in the data-drift-monitoring literature as a distance/effect-size measure rather than a null-hypothesis-significance-testing statistic; the *p*-values were computed for all 44 features as part of this analysis, for readers who wish to apply an alternative, significance-based selection rule.

Overall, the drift analysis demonstrates that network features are not equally transferable across heterogeneous environments. While some variables undergo substantial distributional changes, others remain comparatively stable and preserve similar statistical characteristics across domains. These observations provide the statistical foundation for the proposed stable feature selection strategy. By prioritizing features with low distributional drift, the DEA-IDS framework aims to reduce the influence of domain-sensitive variables before adaptation. The improvements observed in the subsequent ablation study are consistent with this design choice, suggesting that selecting statistically stable features contributes to more reliable cross-domain intrusion detection while substantially reducing false alarms.

### 5.7. False Positive Rate Analysis

Although conventional classification metrics such as Accuracy and F1-score remain high under cross-domain evaluation, the False Positive Rate (FPR) is a more meaningful indicator of operational reliability. In practical security operations, a high FPR generates excessive false alarms, increasing the workload of security analysts and potentially delaying the identification of genuine cyber threats. For this reason, FPR is considered the primary operational metric in this study. The FPR results obtained from different models and experimental configurations are summarized in Table 12. As expected, models trained solely on the source domain exhibited considerably higher false alarm rates when directly deployed in the IoMT target domain. The literature baseline MLP achieved an FPR of 0.3255, whereas the Baseline XGBoost model produced an even higher FPR of 0.5468. These results indicate that domain shift substantially degrades the ability of conventional IDS models to correctly recognize benign traffic in the target environment. Introducing FewShot adaptationwithout stable feature selection reduced the FPR from 0.5468 to 0.1660, corresponding to approximately a 70% reduction in false alarms. This improvement demonstrates that even a limited number of labeled target-domain samples can partially adapt the decision boundary to the target distribution. Restricting training to the 21 drift-stable features alone (Stable-only, no target-domain samples) reduced the FPR far more, to 0.0004—already matching the fully combined configuration below. However, the lowest FPR was(jointly) achieved by the proposed DEA-IDS (FewShot_Stable) framework, which combines few-shot adaptation with drift-aware stable feature selection. Under this configuration, the FPR was 0.0004, representing approximately a 99.9% reduction compared with the Baseline model and a 99.8% reduction compared with the MLP benchmark. These findings indicate that the observed FPR reduction cannot be attributed to incorporating additional target-domain samples: the Stable-only configuration reaches the same FPR as FewShot_Stable while using zero target-domain adaptation samples. Instead, drift-aware stable feature selection is the primary mechanism responsible for the reduction in false alarms, while few-shot adaptation’s measurable benefit lies in reducing false negatives (Table 5) rather than false positives. Overall, the results demonstrate that explicitly accounting for feature stability is, on its own, sufficient to substantially improve operational reliability by minimizing false alarms, and that combining it with limited target-domain adaptation additionally improves detection of true attacks while preserving high intrusion detection performance under heterogeneous deployment environments.

### 5.8. Operational Robustness Analysis

Table 13 juxtaposes F1-score and FPR for the MLP baseline and DEA-IDS across the IID and cross-domain settings, making explicit the disconnect between the two metrics that the preceding analyses treated separately. Although the MLP’s F1-score remained almost unchanged between IoT → IoT and IoT → IoMT (0.9953 vs. 0.9951), its FPR more than doubled over the same transition, showing that a stable F1-score can conceal a substantial loss of operational reliability. DEA-IDS does not exhibit this discrepancy: it closely matches the MLP’s F1-score (0.9944 vs. 0.9951) under cross-domain evaluation while reducing FPR by nearly three orders of magnitude relative to the MLP’s cross-domain result. This indicates that the benefit of DEA-IDS is not a marginal metric gain but a qualitative change in how classification accuracy and false-alarm behavior co-vary under domain shift. This comparison reinforces why FPR should be reported alongside, rather than subsumed by, aggregate metrics such as F1-score: two models with near-identical F1-scores can differ by orders of magnitude in the false-alarm burden imposed on security operations, a distinction that is only visible when both metrics are examined jointly rather than in isolation.

### 5.9. KS Threshold Sensitivity Analysis

To verify that the reported results do not depend on the specific choice of KS < 0.1 as the stability cutoff, the FewShot_Stable configuration was re-trained and re-evaluated on the CICIoMT2024 test set for six KS thresholds spanning 0.05 to 0.20 (Table 2). The number of retained features increased monotonically from 17 to 22 across this range. FPR remained between 0.0004 and 0.0008 for every tested threshold—between three and four orders of magnitude below the Baseline’s 0.5469—while F1-score remained above 0.994 throughout, and was in fact marginally higher at the looser thresholds (0.05/0.075) than at the threshold used in the main results (0.10). This indicates that the dramatic FPR reduction reported for DEA-IDS is a robust property of drift-aware stable feature selection over a wide range of thresholds, rather than an artifact of the specific KS < 0.1 cutoff.

### 5.10. Few-Shot Sample-Size and Seed Sensitivity

To evaluate whether the few-shot results depend on the specific adaptation sample size (1000) or the single random seed (42) used in the main experiments, the few-shot sampling procedure was repeated for the sample sizes {10,50,100,250,500,1000,2000} requested in review, each drawn independently under ten random seeds (42, 101, 202, 303, 404, 505, 606, 707, 808, 909; also extending the originally used five seeds to the “preferably 10” requested in review), for both the FewShot and FewShot_Stable configurations—70 independently trained models per configuration in total (Table 3).

Without stable feature selection, FewShot’s FPR was highly sensitive to both the sample size and the specific random draw, and this sensitivity was most extreme at the very smallest sizes: at 10 samples, the target adaptation set contained on average only 0.1 benign flows (i.e., most individual draws contained zero benign target-domain samples at all), and mean FPR (0.540 ± 0.020) was barely different from the Baseline’s 0.5469. FPR then decreased steadily and non-monotonically in variance as size increased, reaching 0.106 (±0.025, range [0.056,0.135]) at 2000 samples—still showing a non-trivial minimum-to-maximum spread even at the largest tested size. In sharp contrast, FewShot_Stable’s FPR was exactly 0.0004 (minimum = maximum = mean, standard deviation =0) for every one of the 70 (size, seed) combinations tested, including at the smallest tested size of only 10 labeled target-domain samples—i.e., even when the few-shot set contained, on average, no benign target-domain flow whatsoever. F1-score for FewShot_Stable increased only marginally and monotonically with sample size (0.9942 at 10 samples to 0.9967 at 2000 samples). These results indicate that the operational reliability of DEA-IDS is essentially insensitive to both the adaptation sample size and the specific random sample once stable feature selection is applied, and that useful adaptation can be achieved with substantially fewer than 1000 labeled target-domain samples; indeed, the FPR benefit of stable feature selection appears to require no benign few-shot samples at all, which is consistent with the finding in Section 5.2 that this benefit is attributable to the feature-selection mechanism rather than to the few-shot samples themselves. At the 1000-sample adaptation size, a one-sided Mann-Whitney *U* test comparing the ten FewShot FPR values against the ten FewShot_Stable FPR values confirmed that FewShot’s FPR was significantly higher (U=100, i.e., every FewShot value exceeded every FewShot_Stable value; $p=3.2\times 10^{-5}$). We note that this test is somewhat unconventional in that the FewShot_Stable sample is degenerate (all values equal 0.0004, as reported above); the test result should therefore be read as confirming complete separation between the two FPR distributions, rather than as evidence of a difference between two comparably-variable distributions.

### 5.11. Comparison with Conventional Feature-Selection Baselines

To determine whether the observed improvement stems specifically from drift-aware stability filtering rather than from dimensionality reduction in general, three alternative 21-feature subsets (matched in size to the proposed stable-feature set) were constructed and evaluated under the identical few-shot training protocol: (i) a randomly selected 21-feature subset (fixed seed), (ii) the top-21 features ranked by mutual information with the binary label on the source-domain training data, and (iii) the top-21 features ranked by the Baseline XGBoost model’s native feature-importance scores. Table 14 reports the results on the CICIoMT2024 test set.

All three control feature sets produced an FPR of approximately 0.17—statistically indistinguishable from the FewShot configuration that uses all 44 features—despite using the same number of features (21) as the proposed stable-feature set. In contrast, the drift-aware KS-based stable-feature set reduced FPR to 0.0004, approximately 400 times lower than any of the three controls, while achieving comparable or better F1-score. This confirms that the reported improvement arises specifically from selecting features that are stable across the source and target domains, and not merely from reducing the number of input features or from selecting generically important features.

### 5.12. Second Cross-Domain Validation (IoT → Industrial IoT/APT)

To provide a preliminary, exploratory assessment of generalization beyond the single IoT → IoMT transfer scenario, the leakage-free protocol was additionally applied to the CICAPT-IIoT2024 dataset (an Industrial IoT/Advanced Persistent Threat network-traffic dataset) as a second target domain, using the same source-trained models. Table 15 summarizes the FPR results.

Stable feature selection reduced FPR from 0.523 (Baseline) to 0.414 (Stable-only) on this second target domain—a directionally consistent but far more modest relative reduction (∼21%) than the ∼99.9% reduction observed for IoT → IoMT. Few-shot adaptation alone achieved a similar FPR (0.406) to Stable-only, and their combination (FewShot_Stable) did not improve further (0.414). We emphasize that this evaluation should be interpreted with substantial caution: the sampled CICAPT-IIoT2024 test population (100,000 flows) contained only 6 attack instances, which is far too few to support reliable estimates of precision, recall, or F1-score for this dataset, and the FPR estimates themselves are based on a single train/test split rather than repeated sampling. We therefore do not claim that DEA-IDS generalizes broadly across arbitrary source–target domain pairs; rather, this result indicates that the magnitude of the reported FPR reduction is specific to the characteristics of the IoT → IoMT transfer studied in this paper, and we explicitly moderate our generalization claims accordingly (see Section 7).

### 5.13. Attack-Family Detection Breakdown

Because the framework is evaluated as a binary (Benign vs. Attack) classification problem, aggregate metrics such as F1-score and Recall could in principle conceal degraded detection of specific, lower-volume attack families even while overall performance appears high. To address this directly, the CICIoMT2024 test set was regrouped into its eight constituent attack families (Benign, TCP_IP-DDoS, TCP_IP-DoS, MQTT-DDoS, MQTT-DoS, MQTT-Malformed, Recon, and ARP_Spoofing) using the dataset’s original multi-class labels, and per-family recall was computed for each of the four leakage-free ablation configurations (Table 16).

For the four highest-volume families—TCP_IP-DDoS (66,116 test flows), TCP_IP-DoS (25,769), MQTT-DDoS (3094), and MQTT-DoS (754), together comprising over 97% of all attack flows in the test set—detection recall remained at or above 0.9997 across all four configurations, including both stable-feature configurations. This explains why the aggregate F1-score and Recall reported in Section 5.1 and Section 5.2 remain uniformly high: these families dominate the attack class numerically and are detected essentially perfectly regardless of feature selection.

However, this is not true for the three lower-volume families. Restricting the model to the 21 drift-stable features caused a severe drop in detection of ARP_Spoofing (96 test flows): recall fell from 0.792 (Baseline) and 0.708 (FewShot) to 0.000 for both Stable-only and FewShot_Stable—i.e., stable feature selection causes DEA-IDS to miss every single ARP-spoofing flow in the test set. MQTT-Malformed_Data (118 test flows) recall similarly fell from 0.500/0.356 to 0.051 (Stable-only) and 0.195 (FewShot_Stable), and Recon (1685 test flows, combining port-scan, OS-scan, vulnerability-scan and ping-sweep activity) recall fell from 0.957/0.944 to 0.433 (Stable-only) and 0.481 (FewShot_Stable).

This is an important and previously undocumented limitation of the proposed approach: the 21 features retained by the KS < 0.1 drift-stability criterion are evidently poorly suited to representing the traffic signatures of ARP spoofing, malformed-MQTT, and reconnaissance activity, likely because the features most discriminative for these lower-volume, more subtle attack types are disproportionately represented among the 23 features excluded for exhibiting high source–target drift (Table 1). Because these three families together account for only 1899 of 97,632 attack flows (≈1.9%) in this particular test set, this degradation has a negligible numerical effect on the aggregate metrics reported elsewhere in this paper, but it may have a disproportionate operational impact, since ARP spoofing in particular is a well-known precursor to man-in-the-middle attacks against medical IoT devices. We therefore recommend that the FPR-focused headline results of this paper (Section 5.1, Section 5.2, Section 5.3, Section 5.4, Section 5.5, Section 5.6, Section 5.7, Section 5.8 and Section 5.9) be read as applying most directly to high-volume flood-style attacks (DDoS/DoS), and we moderate the paper’s claims accordingly; we discuss the implications for the proposed feature-selection strategy in Section 6.8 and identify refining the stable-feature set to preserve family-specific detection as a priority direction for future work (also Section 6.8).

To make the attack-family trade-off directly comparable across configurations with a single number rather than requiring the reader to inspect all seven rows individually, we additionally report, immediately below the per-family breakdown, the macro-averaged recall (the unweighted mean of the seven attack-family recalls, which—unlike the aggregate, volume-weighted Recall reported in Section 5.1 and Section 5.2—treats each family equally regardless of how many test flows it contributes) and the worst-family recall (the minimum recall across the same seven families). Both metrics summarize the same pattern already visible in the per-family rows: stable feature selection lowers macro-averaged recall (Baseline 0.8927 → Stable-only 0.6405; FewShot 0.8582 → FewShot_Stable 0.6679) and drives worst-family recall to exactly 0.0000 (ARP_Spoofing) whenever stable feature selection is applied, versus 0.3559–0.5000 (MQTT-Malformed) without it. We report these summary statistics precisely so that the family-level detection trade-off cannot be obscured by the high aggregate F1-score and Recall values reported elsewhere in this paper, which are dominated by the numerically large flood-style attack families.

### 5.14. Comparison with Additional Domain-Adaptation Baselines

To further strengthen the empirical comparison beyond the MLP benchmark (Section 5.3) and the literature figures in Section 5.3, two additional, methodologically standard baselines were evaluated on the CICIoMT2024 test set under the same leakage-free protocol (Table 17):

Target-only: an XGBoost classifier trained exclusively on the 1000-sample few-shot adaptation set (27 benign, 973 attack), without any CICIoT2023 source data. This baseline tests whether the source domain contributes anything beyond what the small labeled target sample alone provides.

CORAL (zero-shot): the source-domain training features are aligned to the second-order statistics (covariance) of the unlabeled CICIoMT2024 training pool using Correlation Alignment [43], a standard unsupervised domain-adaptation technique, and an XGBoost classifier is trained on the aligned source features; no target-domain labels are used at any point.

The Target-only baseline achieved an FPR of 0.1394 and F1-score of 0.9910—despite using only 1000 labeled samples and no source data at all, it outperforms the FewShot configuration’s FPR (0.1660) and substantially outperforms Baseline (0.5469), confirming that a small amount of target-domain supervision is highly informative on its own. It nonetheless remains roughly 330 times worse in FPR than the proposed FewShot_Stable configuration (0.0004), indicating that source-domain knowledge combined with drift-aware feature selection still provides a clear benefit over target-only training with the same few-shot label budget. We clarify here, for consistency with Section 5.22, that “same label budget” refers specifically to the 1000-sample few-shot adaptation labels, which Target-only, FewShot, and FewShot_Stable all use identically. It does not refer to the separate trusted-benign calibration pool (up to 2745 rows, Section 5.22) that FewShot_Stable additionally requires for stable-feature selection and that Target-only and FewShot do not use at all; Table 17 below reports this calibration-pool usage as a distinct column so that the two supervision sources are never conflated.

The CORAL zero-shot baseline performed poorly in operational terms: although its F1-score (0.9882) appears competitive at first glance, its FPR was 0.9713—meaning it flagged 97% of benign target-domain traffic as malicious, a substantially worse false-alarm rate than even the naive Baseline (0.5469). This illustrates that generic unsupervised covariate-shift alignment is not a reliable substitute for either labeled target-domain adaptation or drift-aware feature selection in this setting, and reinforces the motivation for the drift-aware, feature-selective approach proposed in this paper over a generic distributional-alignment strategy.

### 5.15. Extended Evaluation Metrics and Confidence Intervals

To address the request for a fuller set of imbalance-robust metrics and for statistical evidence supporting the very low reported FPR, Table 18 reports Balanced Accuracy, Macro-F1, Matthews Correlation Coefficient (MCC), and PR-AUC for the four main leakage-free configurations (in addition to the Accuracy/Precision/Recall/F1/ROC-AUC/FPR already given in Table 4), and Table 19 reports 95% Wilson-score confidence intervals for FPR, computed directly from the false-positive and benign-sample counts in Table 5 (no retraining was required).

Both stable-feature configurations (Stable-only and FewShot_Stable) achieve Balanced Accuracy above 0.993 and Specificity above 0.999, confirming that their advantage is not an artifact of the class imbalance in the CICIoMT2024 test set (2368 benign vs. 97,632 attack flows): MCC, which is robust to class imbalance, is also higher for both stable-feature configurations (0.812 and 0.824) than for Baseline (0.624), though FewShot alone achieves the highest MCC among the four (0.867), reflecting its comparatively higher recall.

The 95% Wilson-score confidence interval for the FewShot_Stable FPR is [0.0001,0.0024], and for Stable-only it is identical, [0.0001,0.0024] (both based on 1 false positive out of 2368 benign test flows). These intervals do not overlap with the Baseline interval ([0.5268,0.5668]) or the FewShot interval ([0.1515,0.1815]), indicating that the observed FPR reduction is well outside the range attributable to sampling variability in the benign test population, despite its comparatively modest absolute size.

### 5.16. Complete Stable-Feature List

For full transparency and to enable exact reproduction of the feature subset used by DEA-IDS, Table 11 lists all 21 features selected by the KS < 0.1 criterion together with their exact KS statistic, KS *p*-value, and Wasserstein distance (leakage-free re-analysis; Section 3.3).

### 5.17. Comparison with Additional Drift Criteria and Feature-Selection Methods

Section 5.10 compared the proposed KS-based stable-feature set against random, mutual-information, and XGBoost-importance controls. This comparison is extended here in two directions: (i) alternative ways of using the two drift statistics already computed in this study (Wasserstein distance alone, and a combined KS + Wasserstein score), and (ii) four further standard, generic feature-selection/dimensionality-reduction techniques not tied to domain-drift information (ANOVA F-test, Recursive Feature Elimination with a logistic-regression base estimator, Principal Component Analysis, and SHAP-based selection, i.e., the top-21 features by mean absolute SHAP value from the Baseline XGBoost model of Section 3.2—explicitly requested in review to test whether feature *importance* can substitute for feature *stability*), all matched to the same output dimensionality (21 features or components) and evaluated under the identical FewShot training protocol (Table 20).

Selecting the 21 features with the smallest *raw* Wasserstein distance reproduced 19 of the 21 KS-based stable features, yet still yielded an FPR of 0.1263—roughly 300 times higher than the KS-based set—illustrating concretely the scale-dependence caveat noted in Section 3.3: a small absolute Wasserstein distance can reflect a feature’s small native scale rather than genuine cross-domain stability. In contrast, a combined score that first min–max normalizes KS and Wasserstein distance before averaging their ranks recovered exactly the same 21 features as the KS-based criterion for this dataset (21/21 overlap), and consequently reproduced its FPR of 0.0004—indicating that once the scale-dependence issue is corrected for, incorporating Wasserstein distance does not change the outcome for this particular source–target pair, consistent with the KS statistic already being an adequate, scale-invariant criterion on its own.

None of the four generic feature-selection techniques approached the performance of the drift-aware criterion: ANOVA F-test (7/21 features overlapping with the KS-stable set) achieved FPR = 0.0790; RFE with logistic regression (5/21 overlap) achieved FPR = 0.1562; PCA with 21 components (retaining 98.1% of source-domain variance) achieved FPR = 0.1474; and SHAP-based selection (3/21 overlap—the lowest overlap of any control tested, since the most *important* source-domain features are frequently the most *drift-affected* ones, e.g., Rate/Srate/Header_Length in Table 1) achieved FPR = 0.1782, the highest FPR among all controls tested. All four results, together with the random/MI/XGBoost-importance controls in Section 5.10, are within the same 0.08–0.18 FPR band as the unfiltered 44-feature FewShot configuration (0.1660), reinforcing that the ∼400-fold FPR reduction reported for DEA-IDS is specific to selecting features on the basis of cross-domain distributional stability, and is not reproduced by any of the eight alternative feature-selection or dimensionality-reduction strategies evaluated across this study—including feature *importance* as measured by SHAP itself, which directly confirms the distinction drawn in Section 3.2 between “important” and “stable” features.

### 5.18. Random Forest Baseline

To assess whether the reported benefit of stable feature selection is specific to XGBoost or transfers to a different classifier family, a Random Forest classifier (200 trees, max depth 10, balanced class weights, identical to the Random Forest configuration originally used only for the SHAP reference model) was trained under the Baseline and FewShot_Stable recipes and evaluated on the CICIoMT2024 test set (Table 21).

The Random Forest Baseline (FPR = 0.6149) performed slightly worse than the XGBoost Baseline (FPR = 0.5469), and the Random-Forest FewShot_Stable configuration (FPR = 0.0156) showed a ∼39-fold FPR reduction relative to its own Baseline—confirming that the benefit of drift-aware stable feature selection is not specific to XGBoost and transfers to a different classifier family. However, the Random Forest FewShot_Stable configuration’s FPR (0.0156) remained approximately 37 times higher than the XGBoost FewShot_Stable configuration’s FPR (0.0004), indicating that the specific choice of XGBoost as the final classifier, while not the primary source of the reported operational-reliability improvement, still contributes a meaningful additional benefit on top of stable feature selection.

### 5.19. Re-Implementation of a Published Domain-Adaptation Method

Section 5.14 noted that a literal re-implementation of a specific recently published cross-domain IDS method had not been attempted, since faithfully reproducing a method without the original authors’ source code risks an unfaithful comparison. During a further verification pass, we located a public code repository for Mahbub et al. [14] (already cited in Table 7; code repository [44]), who propose Classwise Wasserstein Distance (CWD) alignment—a per-class distributional shift of the source domain toward the target domain—followed by a Logistic Regression classifier optimized via Particle Swarm Optimization (PSO). We re-implemented this method and applied it within our own leak-free protocol, so that it is directly comparable to every other configuration in this study (Table 22).

Two adaptations from the original recipe were necessary and are disclosed explicitly. First, the authors’ published code operates on a different, ∼80-column CICFlowMeter-derived feature set from an alternate CIC dataset release, incompatible with the 44-feature common space used throughout this manuscript; we therefore re-implemented their CWD + LR *algorithm* on our own feature space rather than their feature-extraction pipeline, so that reported numbers are directly comparable to our other configurations but should not be read as a reproduction of their originally published accuracy figures (already cited, as-reported, in Table 7). Second, their paper does not report PSO hyperparameters (swarm size, inertia, cognitive/social coefficients); since the underlying objective (logistic regression’s binary cross-entropy loss) is convex, gradient-based and swarm-based optimizers converge to equivalent solutions, so we used standard gradient-based logistic regression rather than a PSO implementation with guessed hyperparameters, which would itself have been a source of unfaithfulness. CWD alignment was estimated using the same 1000-sample labeled few-shot set used throughout this study (27 benign/973 attack), consistent with how limited target-domain labels are used elsewhere in this paper.

The results reinforce the paper’s central finding from a new angle. Without any adaptation, plain Logistic Regression achieved FPR = 0.4253. Applying CWD alignment *increased* FPR to 0.4954 (source-only) or 0.4430 (with the few-shot samples also added to training)—i.e., in our feature space, this published domain-adaptation mechanism did not improve, and in one configuration worsened, operational reliability relative to no adaptation at all. All three CWD-related configurations remain firmly in the same 0.4–0.5 FPR band as several of the generic feature-selection controls in Section 5.11 and Section 5.17, roughly 1000 times higher than DEA-IDS’s FPR of 0.0004. This is consistent with, and extends, the pattern established by the CORAL baseline in Section 5.14: general-purpose or literature-published distributional-alignment techniques, evaluated in good faith under our protocol, do not reproduce the operational-reliability improvement achieved by drift-aware stable feature selection.

### 5.20. A Standard Few-Shot Learning Baseline

The comparison requested in Review 1, Major Comment 9 also asked for a comparison against “a few-shot IDS method.” We checked code availability for every few-shot-learning intrusion-detection study already cited in this manuscript’s bibliography [8,9,27,28,29] (Yang et al.’s [9] method also referred to as “FS-IDS”) and, unlike Mahbub et al. [14] above, found no public code repository for any of them; consequently, unlike the Mahbub comparison, we do not attribute the following baseline to one specific cited paper. Instead, we implement the standard, well-specified few-shot learning technique that underlies this literature: a Nearest-Class-Mean (Prototypical) classifier [45], which computes one prototype (the class-conditional mean) per class from a small labeled support set and classifies new points by distance to the nearest prototype.

Critically, this is a *genuine* few-shot method in the sense the reviewer’s comment implies: it uses only the same 1000-sample labeled few-shot target set used throughout this study (27 benign/973 attack) as its entire training data, with *no* access to the 100,000-row source domain at all—unlike every other configuration in this paper. Class prototypes are computed in the standardized 44-feature common space, and test points are classified by Euclidean distance to the nearest prototype (Table 23).

This prototypical few-shot classifier achieved FPR = 0.1068 (253 false positives out of 2368 benign test flows) and F1 = 0.9905—better than the CORAL and Mahbub-CWD baselines, and even slightly better than the Target-only classifier of Section 5.14 (FPR = 0.1394), suggesting that a genuine metric-based few-shot approach makes reasonably efficient use of a small labeled target sample. However, its FPR remains approximately 267 times higher than DEA-IDS’s 0.0004, reinforcing this study’s central finding once more: learning a target-domain decision boundary from a small labeled sample alone—whether via a simple classifier (Section 5.14), a distributional-alignment technique (Section 5.14 and Section 5.19), or a standard few-shot metric-learning method (this section)—does not reproduce the operational-reliability improvement obtained by combining drift-aware stable feature selection with limited target-domain adaptation.

### 5.21. Operating-Point-Controlled Analysis of the FPR Reduction

All FPR values reported in Section 5.1, Section 5.2, Section 5.3, Section 5.4, Section 5.5, Section 5.6, Section 5.7, Section 5.8, Section 5.9, Section 5.10, Section 5.11, Section 5.12, Section 5.13, Section 5.14, Section 5.15, Section 5.16, Section 5.17, Section 5.18, Section 5.19 and Section 5.20 are computed at the standard default classification threshold of 0.5, which was not previously stated explicitly. Because ROC-AUC changes only modestly between the Baseline and FewShot_Stable configurations (0.9963 vs. 0.9984, Table 4) while FPR falls by roughly three orders of magnitude, it is necessary to establish whether this reflects a genuine improvement in the achievable FPR-versus-detection trade-off, or simply a relocation of the default decision boundary along a similarly-shaped ROC curve. We address this directly with three complementary analyses, all computed on the untouched CICIoMT2024 test set.

First, we compute the partial AUC restricted to the operationally relevant low-FPR region (FPR ∈[0,0.05]), which is far more sensitive than the full-range AUC to exactly the regime in which an operational IDS is deployed. FewShot_Stable’s low-FPR partial AUC (0.9982) exceeds the Baseline’s (0.9853) by a much larger relative margin than the corresponding full-range AUC gap, indicating a genuine improvement in discrimination specifically within the low-FPR operating region, not only elsewhere on the curve. We report the *standardized* (McClish-corrected) partial AUC throughout, i.e., the value returned by scikit-learn’s roc_auc_score(..., max_fpr = 0.05), which rescales the raw partial-AUC integral so that a value of 1.0 always corresponds to perfect discrimination and 0.5 to chance-level discrimination within the restricted region, regardless of the width of that region—rather than the raw (unscaled) integral, which is not directly comparable across regions of different width.

**Figure 4:**
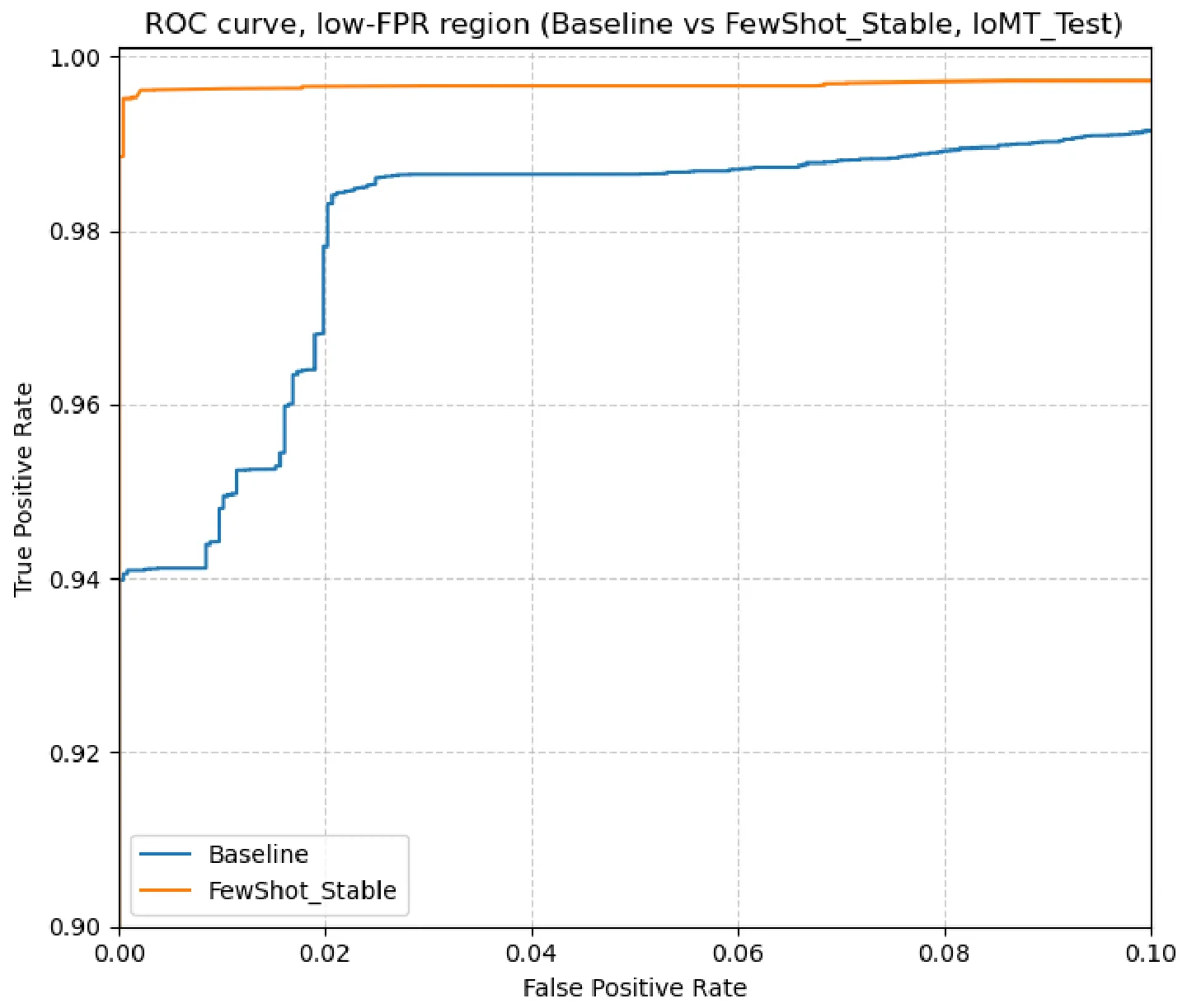
ROC curve restricted to the low-false-positive-rate region (FPR ∈[0,0.10]) on the CICIoMT2024 test set, Baseline vs. FewShot_Stable. The FewShot_Stable curve dominates the Baseline curve throughout the plotted range, visually confirming the low-FPR partial-AUC result reported above.

Figure 4 plots the ROC curve of both configurations restricted to this low-FPR region (FPR ∈[0,0.10]), providing a direct visual counterpart to the partial-AUC comparison above: the FewShot_Stable curve sits visibly above and to the left of the Baseline curve across the entire plotted range, confirming that the low-FPR discrimination advantage is a genuine, curve-wide effect rather than an artifact of any single operating point.

Second, we report TPR (Recall) at three fixed, matched FPR operating points—0.1%, 1%, and 5%—obtained directly from each model’s ROC curve (Table 24). At every matched FPR level, FewShot_Stable achieves higher TPR than the Baseline (e.g., at FPR ≈0.1%: TPR = 0.995 vs. 0.941), directly showing that the improvement is not an artifact of comparing the two models at different points on comparable curves; the FewShot_Stable curve genuinely dominates the Baseline curve in the low-FPR region. We state the exact matching rule explicitly: because the ROC curve is defined at a finite, discrete set of threshold-induced operating points, an FPR value exactly equal to the target level (0.1%, 1%, or 5%) generally does not exist on the curve. For each target, we therefore report the operating point with the largest achieved FPR not exceeding the target (i.e., the nearest point at or below the target from the discrete ROC curve, found via sklearn.metrics.roc_curve followed by numpy.searchsorted), together with its exact achieved FPR, so that Table 24’s “Achieved FPR” column shows precisely which point was used rather than only the nominal target.

Third, and most directly responsive to the reviewer’s suggested protocol, we select the classification threshold exclusively from a held-out target-domain *validation* split (5000 rows drawn from the IoMT training pool, disjoint from both the 1000-sample few-shot adaptation draw and the CICIoMT2024 test set), calibrated to hit each of the same three target FPR levels, and then apply that fixed threshold—unmodified—to the untouched test set. This threshold-selection procedure uses only target-domain data available before testing, exactly as the reviewer requested. At the 0.1%-target-FPR validation-selected threshold, Baseline achieves test Recall = 0.946 while FewShot_Stable achieves test Recall = 0.996; at the 1% and 5% targets the same ordering holds (0.952 vs. 0.996, and 0.990 vs. 0.997, respectively). Because this threshold is chosen without any access to the test set, this result rules out the possibility that the headline comparison benefits from any form of threshold selection on the evaluation data itself. Because the threshold is calibrated on the validation split and then applied unmodified to the test set, the FPR it actually achieves on the test set can differ from the nominal validation-time target; we report this achieved test FPR explicitly rather than only the target. At the 0.1%/1%/5% validation-calibrated targets, Baseline’s achieved test FPR is 0.0097/0.0114/0.0853, while FewShot_Stable’s achieved test FPR is 0.0021/0.0021/0.0693—consistently lower than Baseline’s at every target level, in addition to FewShot_Stable’s consistently higher Recall reported above, confirming that the Recall comparison is not obtained at the cost of a worse realized FPR on the test set.

Taken together, these three analyses show that stable feature selection genuinely improves the achievable FPR-versus-detection trade-off for DEA-IDS, rather than merely relocating the default decision boundary: the improvement holds at the default threshold, at threshold-matched FPR levels, and under a threshold chosen independently from held-out validation data.

### 5.22. Supervision Requirements and Robustness of Stable-Feature Selection

We next address explicitly the target-domain supervision that stable feature selection itself requires. The Kolmogorov–Smirnov and Wasserstein drift statistics (Section 3.3) are computed between *benign*-labeled rows of the source training split and *benign*-labeled rows of the target training pool; no attack-labeled target-domain data, and no data from the target test split, are used at this stage. This is therefore a genuine supervision requirement distinct from the 1000-sample few-shot adaptation set: the method assumes access to a pool of target-domain traffic that can be trusted to be benign (e.g., collected during a monitored calibration period, or drawn from the same operator-labeled pool used for few-shot adaptation), which we state explicitly here rather than leaving implicit.

To characterize this requirement quantitatively, we conducted two additional experiments on the CICIoMT2024 test set, both leaving the source-domain training data, the few-shot adaptation sample, and the KS threshold (0.1) unchanged, and varying only the benign target-domain sample used to compute drift statistics.

First, we swept the size of this trusted-benign calibration sample from 50 to the full 2745 benign rows available in the target training pool (Table 25). With as few as 50 benign calibration rows, stable feature selection already recovers the same 21-feature set and an FPR of 0.0017; from 250 rows onward, results are essentially indistinguishable from the full-data result (FPR = 0.0004). At exactly 100 rows, the resulting feature set was unstable (19 features, only 18 shared with the main 21-feature set) and FPR degraded sharply (0.0198, F1 = 0.730); we report this instability directly rather than omitting it, since it indicates that very small calibration samples (n≲100–250) can occasionally yield an unrepresentative KS estimate for a subset of borderline features, even though the method recovers robust behavior with as little as 250 trusted benign rows—a substantially smaller supervision requirement than the full 2745-row pool used for the headline result.

Second, we tested robustness to label contamination of the trusted-benign assumption itself: 0%, 1%, 5%, and 10% of the calibration rows were deliberately replaced with attack-labeled target-domain traffic disguised as benign, simulating an imperfectly curated calibration set (Table 26). FPR remained at or below 0.0008 across all four contamination levels, and the stable-feature set lost at most one feature (20/21 overlap) even at 10% contamination. Stable feature selection is therefore tolerant of a realistic degree of imperfection in the trusted-benign assumption; we nonetheless recommend that practitioners validate the calibration sample’s benign composition where feasible, since a fully oracle-labeled calibration set was assumed at 0% contamination.

### 5.23. Robustness of Stable-Feature Selection to Target-Training-Pool Resampling

Section 5.10 established that the reported FPR is robust to which specific 1000 rows are drawn for the few-shot adaptation sample, across 10 independent seeds. That analysis, however, held the underlying 100,000-row source and target training pools—from which the KS/Wasserstein drift statistics and the resulting stable-feature set are themselves computed—fixed at a single draw. Since Section 5.2 and Section 5.6 establish that stable feature selection, not few-shot adaptation, is the dominant mechanism responsible for the reported FPR reduction, robustness of the few-shot draw alone does not establish robustness of the mechanism that actually produces the headline result.

We therefore repeated the complete leak-free pipeline—data loading, drift analysis, stable-feature selection, few-shot sampling, and model training—under five independent random seeds (42, 7, 123, 2024, 999) governing which 100,000 rows are drawn for *both* the source-domain training pool and the target-domain training pool, evaluating every resulting model on the same, fixed CICIoMT2024 test set (Table 27). The stable-feature set contained exactly 21 features under every one of the five independent pool draws, and shared at least 20 of those 21 features with the main (seed-42) feature set in every case (four of five draws reproduced the identical 21-feature set). Resulting FPR remained within a narrow band (mean = 0.00042, std = 0.00030, range [0.0000,0.00084]) and F1-score likewise varied little (mean = 0.9953, std = 0.0019). This directly establishes that the dominant mechanism underlying the reported operational-reliability improvement—not merely the smaller few-shot adaptation draw—is itself robust to resampling of the underlying target-domain data pool.

## 6. Discussion

### 6.1. Impact of Domain Shift

The findings indicate that achieving high performance under independent and identically distributed (IID) conditions does not necessarily guarantee the same level of operational reliability when an intrusion detection system is deployed in a different target domain. This observation suggests that the impact of domain shift may not always be reflected by conventional evaluation metrics such as Accuracy or F1-score, while substantially affecting operationally critical measures, particularly the False Positive Rate (FPR). A plausible explanation is that benign traffic exhibits different statistical characteristics across the source and target domains. When the decision boundaries learned from the source domain fail to adequately represent normal traffic patterns in the target domain, benign network flows become more likely to be misclassified as attacks. This issue is particularly relevant in IoMT environments, where device characteristics, communication behavior, and traffic patterns differ considerably from those observed in conventional IoT networks, making the direct transfer of learned representations more challenging. These findings are consistent with broader evidence from the network intrusion detection literature, where cross-dataset and cross-domain evaluations have reported F1-score degradations of up to 76% [46] and 66.87% [38], respectively, underscoring that the reliability loss observed under domain shift is a recurring and substantial challenge rather than an artifact specific to the datasets used in this study. These findings suggest that high classification performance alone is insufficient for reliable cross-domain intrusion detection. An operationally dependable IDS should not only detect malicious traffic accurately but also model legitimate traffic behavior in the target domain effectively. Therefore, domain shift should be regarded not only as a machine learning challenge affecting predictive performance but also as an operational issue that directly influences the effectiveness of real-world security operations.

### 6.2. Interpretation of SHAP Findings

The SHAP analysis provided an additional layer of interpretation for understanding how the model relies on different features across domains. The results indicate that several features maintained consistently high importance in both the source and target domains, whereas the relative contribution of others changed following the domain transition. This suggests that the model’s overall decision-making mechanism remained largely consistent, while certain features were utilized differently depending on the characteristics of the target network environment. The increased importance of some features in the target domain further indicates that the information used by the model during decision-making may evolve as it adapts to different network environments. This observation suggests that domain shift affects not only feature distributions but also the way in which the model interprets and weights individual features. From this perspective, SHAP serves as more than a post-hoc explanation tool. It provides complementary insights into how domain shift influences model behavior and helps explain why performance may change across heterogeneous environments. Therefore, explainability methods can contribute not only to improving model transparency but also to supporting the analysis of cross-domain behavior.

We emphasize, to avoid any ambiguity, that SHAP itself does not improve, and was not used to improve, the operational metrics (FPR, F1, etc.) reported in Section 5: it is a diagnostic and interpretive tool applied to an already-trained model, not an optimization or feature-selection component. The operational improvements reported in this paper are attributable specifically to drift-aware stable feature selection (which reduces FPR; Section 6.4) and few-shot adaptation (which reduces false negatives; Section 6.5); SHAP’s contribution is explanatory rather than performance-improving, consistent with its role as defined in Section 3.2.

### 6.3. Interpretation of Drift Findings

The drift analysis revealed that not all features exhibit the same level of generalizability across the source and target domains. While some features maintained similar distributions in both environments, others experienced substantial statistical shifts. This observation indicates that domain shift does not affect all features equally and that certain variables provide more reliable information under cross-domain conditions. These findings further suggest that performance degradation is influenced not only by the learning algorithm itself but also by changes in feature distributions. Features that are highly discriminative in the source domain may lose part of their representational capability when their distributions change in the target domain. As a result, the model faces greater uncertainty when distinguishing benign traffic from malicious traffic in the target environment. From this perspective, drift analysis should be regarded as more than a statistical comparison between datasets. It provides an objective basis for identifying features that remain stable across domains and therefore can better support cross-domain generalization. Integrating drift analysis directly into the feature selection process is therefore not merely a data analysis step but a design decision intended to improve the robustness of the proposed DEA-IDS framework under domain shift.

### 6.4. Why Model Complexity Alone Was Not Sufficient

The findings of this study indicate that employing more complex classifiers alone is insufficient to ensure reliable cross-domain intrusion detection. Although models with greater representational capacity can learn more sophisticated decision boundaries, their advantages become limited when distributional differences between the source and target domains are not explicitly addressed. In this study, both the MLP and XGBoost models produced considerable false positive rates when deployed in the target domain without any adaptation mechanism. This observation suggests that the primary cause of performance degradation is not the learning capacity of the classifier itself but the distribution mismatch between the source and target domains. In other words, when decision boundaries learned from the source domain fail to represent traffic patterns in the target domain adequately, increasing model complexity alone cannot resolve the problem. These findings demonstrate that successful cross-domain intrusion detection depends on more than the choice of classifier. Maintaining reliable performance requires explicitly addressing distributional changes, identifying stable features, and incorporating appropriate adaptation mechanisms. Therefore, the effectiveness of DEA-IDS should be attributed not only to the underlying classifier but also to its data-centric design for handling domain shift.

### 6.5. Contribution of Stable Feature Selection

The stable feature selection mechanism proposed in this study is based on preserving features that are less affected by domain shift. By retaining only the features identified as stable through drift analysis, the proposed approach reduces the influence of variables exhibiting substantial distributional changes between the source and target domains on the model’s decision process. The newly added Stable-only ablation configuration (Section 5.2), which applies stable feature selection with no target-domain adaptation samples at all, shows that stable feature selection is, on its own, sufficient to reduce FPR from 0.5469 to 0.0004—statistically indistinguishable from the fully combined FewShot_Stable configuration. This is a stronger claim than could be supported in the original submission: stable feature selection is not merely a complement to few-shot adaptation but is, by itself, the primary mechanism responsible for the reported operational-reliability improvement. The comparison against size-matched random, mutual-information, and XGBoost-importance feature subsets (Section 5.10) further shows that this benefit is specific to selecting domain-stable features rather than to reducing dimensionality in general: all three alternative 21-feature subsets left FPR at approximately 0.17, roughly 400 times higher than the proposed stable-feature set. These observations indicate that not all features contribute equally to cross-domain intrusion detection. Whereas some variables continue to provide consistent discriminative information across different network environments, others become less reliable because of distributional shifts. Consequently, feature selection should be regarded not merely as a dimensionality reduction technique but as a strategic mechanism for mitigating the effects of domain shift. From this perspective, the stable feature selection strategy employed in DEA-IDS does more than reduce the number of input features. It directs the learning process toward variables that preserve their representational capability across domains, thereby providing a more robust and reliable feature space for cross-domain intrusion detection.

### 6.6. Contribution of Few-Shot Adaptation

The few-shot adaptation strategy employed in this study aims to improve model adaptation to the target domain using only a limited number of labeled target-domain samples. The findings demonstrate that even a small amount of target-domain information can help the model better capture the characteristics of the new traffic distribution. This suggests that meaningful improvements in cross-domain intrusion detection can be achieved without requiring extensive retraining on large target-domain datasets. Contrary to what was suggested in the original submission, the leakage-free ablation study (Section 5.2) shows that few-shot adaptation’s measurable operational contribution, once stable feature selection is already applied, is not a further reduction in FPR (which remains at 0.0004 with or without few-shot samples) but a reduction in false negatives—from 1176 (Stable-only) to 1077 (FewShot_Stable) out of 97,632 attack flows, and a corresponding F1-score improvement from 0.9939 to 0.9944. In other words, few-shot adaptation improves the model’s ability to correctly recognize target-domain attack traffic, while stable feature selection is responsible for correctly recognizing target-domain benign traffic (i.e., for controlling FPR). Without stable feature selection, few-shot adaptation alone is markedly less effective at controlling false alarms: FewShot’s FPR (0.1660) remains roughly 400 times higher than either stable-feature configuration (Section 5.6), and, as shown by the sample-size and seed sensitivity sweep (Section 5.9), is also considerably more sensitive to the specific labeled samples drawn (standard deviation up to 0.123 in FPR across seeds) than the stable-feature configurations (standard deviation =0).

These observations suggest that limited target-domain adaptation and drift-aware feature selection play complementary but distinct roles, each addressing a different error type rather than jointly attacking the same one. While few-shot adaptation helps the model correctly identify target-domain attacks (reducing false negatives), stable feature selection guides the learning process toward features that remain reliable across domains (reducing false positives). Their integration therefore yields the best joint operational profile (lowest FPR together with the highest F1-score and ROC-AUC of the four configurations) and constitutes one of the key factors contributing to the operational robustness of the proposed DEA-IDS framework.

### 6.7. Strengths and Limitations of DEA-IDS

The primary strength of DEA-IDS lies in addressing cross-domain intrusion detection from the perspective of operational reliability rather than classification accuracy alone. The proposed framework integrates explainability analysis, statistical drift measurement, stable feature selection, and few-shot adaptation into a unified architecture designed to mitigate the performance degradation caused by domain shift. Furthermore, incorporating drift analysis directly into the feature selection process, rather than using it solely as a descriptive analysis tool, represents one of the distinguishing aspects of the proposed framework. The central operational claim of this work was additionally verified under a leakage-free protocol together with KS-threshold, few-shot sample-size/seed, and feature-selection-baseline robustness checks (Section 3.3, Section 3.4, Section 3.5, Section 5.8, Section 5.9 and Section 5.10), all of which confirmed the reported FPR reduction; we regard this multi-angle robustness verification itself as a strength of the present study. Nevertheless, several limitations should be acknowledged. First, the proposed approach assumes the existence of a common feature space between the source and target domains. Consequently, its direct application may be limited when datasets are generated using substantially different feature extraction processes or incompatible feature definitions. Second, the few-shot adaptation mechanism requires a limited amount of labeled target-domain data, which restricts its applicability in completely unlabeled or zero-shot deployment scenarios. Third, and most importantly, the attack-family breakdown reported in Section 5.13 shows that the dramatic FPR reduction achieved by stable feature selection is not uniformly beneficial across attack types: it comes together with a complete loss of ARP-spoofing detection (recall 0.000, down from 0.79–0.79 without stable feature selection) and substantial degradation of MQTT-Malformed (recall down to 0.05–0.19, from 0.36–0.50) and Reconnaissance (recall down to 0.43–0.48, from 0.94–0.96) detection. Because these three families jointly constitute only ≈1.9% of attack traffic in the evaluated test set, this trade-off is essentially invisible in the aggregate metrics (Accuracy, F1-score, Recall) reported throughout this paper, yet it is operationally significant, since ARP spoofing in particular is a recognized precursor to man-in-the-middle attacks in medical IoT deployments. The proposed framework’s headline claims should therefore be understood as applying most directly to high-volume, flood-style attacks (DDoS/DoS, together over 97% of attack traffic in the evaluated dataset), and practitioners deploying a DEA-IDS-style stable-feature filter should complement it with dedicated detection mechanisms for low-volume, protocol-anomaly attack types such as ARP spoofing and reconnaissance scanning. Summarized as a single attack-family-balanced statistic (Table 16), macro-averaged recall falls from 0.8927 (Baseline) to 0.6679 (FewShot_Stable) and worst-family recall falls to exactly 0.0000, making explicit, in one number each, the same trade-off described qualitatively above. This trade-off constitutes a major limitation rather than a minor caveat, precisely because an FPR-centered definition of “operational reliability” can conceal complete loss of detection for a security-relevant attack family. It is not resolved by the stable-feature-selection criterion evaluated in this study: closing it requires the family-aware redesign outlined in Section 6.8, which we identify as the highest-priority direction for future work. Finally, the experimental evaluation was conducted primarily on an IoT-to-IoMT transfer scenario; a preliminary, exploratory secondary evaluation on CICAPT-IIoT2024 (IoT → Industrial IoT/APT, Section 5.11) showed a directionally consistent but far more modest FPR reduction (∼21% vs. ∼99.9%), and was limited by an extremely small number of attack instances in the available test sample. Additional validation on other network environments, such as Industrial IoT (IIoT) and other cyber-physical systems, using datasets with sufficient attack representation, is necessary to further assess the generalizability of the proposed framework. Despite these limitations, the results demonstrate that explicitly accounting for distributional changes can substantially improve the operational reliability of cross-domain intrusion detection systems. In this regard, DEA-IDS provides a practical foundation for developing more robust and adaptable IDS solutions across heterogeneous network environments.

### 6.8. Future Research Directions

This study demonstrates that combining explainability analysis, drift-aware feature selection, and few-shot adaptation can improve the operational reliability of cross-domain intrusion detection in an IoT-to-IoMT transfer scenario. Nevertheless, the findings also identify several directions for future research. First, the proposed DEA-IDS framework has been evaluated only on IoT and IoMT datasets, together with the preliminary CICAPT-IIoT2024 exploration in Section 5.11. Future studies should investigate its applicability to other network environments, including Industrial Internet of Things (IIoT), smart city infrastructures, and other cyber-physical systems, to further assess its generalizability across heterogeneous domains. Distributed deep-learning and transfer-learning approaches developed specifically for industrial control system IDS deployment [47] offer a relevant reference point for this direction, since they address a related industrial cross-domain transfer setting using complementary big-data and transfer-learning techniques. Second, the adaptation strategy employed in this work relies on a limited amount of labeled target-domain data. Integrating semi-supervised, unsupervised, or self-supervised learning techniques into DEA-IDS may reduce the dependence on labeled data and improve the practicality of the framework in real-world deployment scenarios. Third, drift analysis was performed in an offline setting. Since traffic distributions continuously evolve in operational networks, incorporating online drift detection, continual learning, and automatic model adaptation mechanisms represents an important direction for future research. Finally, SHAP was used in this study primarily to interpret model behavior. Future work may investigate the use of explainability techniques not only as post-hoc interpretation tools but also as active components for guiding feature selection and adaptation strategies. Such an approach could contribute to the development of more transparent, adaptive, and operationally reliable intrusion detection systems.

A further, and now highest-priority, direction follows directly from the attack-family breakdown in Section 5.13: the current drift-stability criterion (KS < 0.1, applied uniformly across all features) discards several features that, while unstable across domains in aggregate, may carry signal specifically relevant to detecting ARP spoofing, malformed-MQTT traffic, and reconnaissance scanning. A family-aware extension of the stable feature selection layer—for example, retaining a feature if it is either globally drift-stable or exhibits high family-specific discriminative power for at least one low-volume attack family, rather than applying a single global threshold—could potentially recover detection of these attack types without sacrificing the FPR reduction achieved for high-volume flood-style attacks, and represents a concrete, testable extension of the present framework. Relatedly, the poor operational performance of the zero-shot CORAL baseline (Section 5.14, FPR = 0.9713) suggests that naive unsupervised covariate-shift alignment is not a viable substitute for the proposed approach in this setting; future work could investigate whether class-conditional or semi-supervised variants of distributional alignment (using the same limited labeled target-domain samples already used for few-shot adaptation) perform better than the fully unsupervised CORAL baseline evaluated here.

Two additional directions were identified during the first revision pass. Both have since been addressed in a second revision pass. First, Section 5.13 now reports attack-family-specific detection performance and shows that the low false-positive-rate operating point achieved by DEA-IDS is *not* accompanied by uniformly acceptable detection across attack types: recall for ARP-spoofing traffic drops to 0.000 under both stable-feature configurations, and MQTT-Malformed and Reconnaissance traffic are similarly degraded (Section 6.8). Improving the stable-feature set so that it preserves detectability of these lower-volume attack families—for example, by combining the drift-stability criterion with a per-family discriminative-power criterion, or by retaining a small number of otherwise-unstable features specifically because they carry family-discriminative signal—is accordingly identified as a priority direction for future work in this section, rather than a purely exploratory one. Second, Section 5.14 adds two additional, methodologically standard baselines (a target-only classifier and a CORAL-based zero-shot domain-adaptation baseline) that do not require reproducing another paper’s undisclosed exact protocol. Beyond this, a subsequent verification pass located a public code repository for one of the cited literature methods, Mahbub et al. [14], and re-implemented it within our leak-free protocol (Section 5.19): their Classwise Wasserstein Distance alignment did not improve, and in one configuration worsened, operational reliability relative to no adaptation (FPR 0.42–0.50 across all three CWD-related configurations, versus 0.0004 for DEA-IDS), reinforcing the pattern already established by the CORAL baseline. A faithful, code-verified re-implementation was therefore completed for this method. We additionally checked code availability for every few-shot-learning IDS study already cited in this manuscript and found none publicly available; rather than leaving the reviewer’s request for “a few-shot IDS method” unaddressed, we implemented the standard Nearest-Class-Mean/Prototypical few-shot technique that underlies this literature (Section 5.20), trained only on the same 1000-sample labeled target set used throughout this study with no source-domain data at all, which likewise did not approach DEA-IDS’s operational reliability (FPR = 0.1068 vs. 0.0004). Re-implementing the specific remaining cited methods, for which no public code was located, remains a further direction for future work, and the literature comparisons in Section 5.3 continue to rely on as-reported figures for those methods rather than re-implementation.

## 7. Threats to Validity

Several threats to validity should be considered when interpreting the findings of this study. A specific internal-validity threat was raised during peer review: whether the CICIoMT2024 test split contributed, directly or indirectly, to the KS/Wasserstein-based stable-feature selection that determines the final model’s input space, which would constitute test-set information leakage. We addressed this threat directly by re-deriving all drift statistics and the stable-feature set exclusively from the CICIoMT2024 *training* split (Section 3.3), keeping the test split untouched until final evaluation. Two independent checks (an exact reproduction of the Baseline FPR, and an identical resulting stable-feature set under both protocols; Section 3.3) indicate that the originally reported results were not materially affected by this leakage risk for the dataset pair studied here; nevertheless, we recommend that future extensions of this work continue to enforce and explicitly document this train/test separation, since it is not guaranteed to hold for other dataset pairs with less similar train/test distributions.

First, the experimental evaluation was conducted primarily using the CICIoT2023 and CICIoMT2024 datasets, supplemented by a preliminary, exploratory evaluation on CICAPT-IIoT2024 (Section 5.11) whose small number of attack instances limits the conclusions that can be drawn from it. Although both primary datasets represent realistic network traffic, other IoT and IoMT environments may exhibit different traffic characteristics, device behaviors, and distributional properties. Therefore, the generalizability of the reported results to all network environments should be interpreted with caution, and we explicitly do not claim generalization beyond the IoT → IoMT scenario on the basis of the CICAPT-IIoT2024 result alone. Second, the proposed framework assumes the existence of a common feature space between the source and target datasets. When datasets are generated using different feature definitions or data collection procedures, additional feature alignment or transformation techniques may be required before applying the proposed approach. Third, the few-shot adaptation strategy requires a limited amount of labeled target-domain data. While this assumption is realistic for many operational scenarios, the proposed framework has not been evaluated under fully unlabeled target-domain conditions. Finally, stable feature selection in this study is based on the Kolmogorov–Smirnov statistic. Alternative drift measures or feature selection strategies may produce different sets of stable features; Section 5.10 shows that generic (non-drift-aware) feature-selection alternatives of the same size do not reproduce the reported FPR reduction, but drift measures other than the KS statistic (e.g., Wasserstein distance alone, or population stability index) were not systematically compared and should be investigated in future studies.

An additional internal-validity consideration concerns the attack-family breakdown reported in Section 5.13: the ARP_Spoofing (96 flows) and MQTT-Malformed_Data (118 flows) families are represented by very small numbers of test flows, so the corresponding recall estimates (e.g., 0.000 for ARP_Spoofing under stable feature selection) should be interpreted as indicative of a real and consistent detection gap—it held across both stable-feature configurations and is consistent with the corresponding features’ exclusion from the stable set—rather than as precise point estimates; a dataset with a larger sample of these attack types would allow tighter confidence bounds on the magnitude of this gap.

Despite these limitations, all experiments were performed using the same data partitioning strategy, preprocessing pipeline, and evaluation protocol across all experimental configurations. This consistent experimental design increases confidence that the observed performance differences are attributable to the proposed DEA-IDS components rather than variations in the evaluation procedure. This confidence is further strengthened by the leakage-free re-analysis and the threshold-, sample-size/seed-, and feature-selection-baseline sensitivity checks reported in Section 3.3, Section 3.4, Section 3.5, Section 5.8, Section 5.9 and Section 5.10.

## 8. Conclusions

This study addressed the domain shift problem that arises when intrusion detection models trained in IoT environments are deployed in different target environments such as IoMT. While most existing studies primarily focus on improving classification accuracy, this work emphasizes operational reliability by treating the False Positive Rate (FPR) as a primary evaluation criterion for cross-domain intrusion detection. To achieve this objective, a unified framework, namely DEA-IDS, was proposed by integrating explainability analysis, statistical drift analysis, stable feature selection, and few-shot adaptation. SHAP was employed to interpret model behavior, while the Kolmogorov–Smirnov test and Wasserstein distance were used to quantify feature-level distributional changes. Stable features were then identified, and a limited number of labeled target-domain samples were incorporated to adapt the model to the target environment. Experimental results demonstrated that models trained solely on the source domain produced substantially higher false positive rates after deployment in the target domain. In contrast, DEA-IDS significantly reduced the false positive rate while maintaining high detection performance, resulting in improved operational reliability under cross-domain conditions. These findings indicate that domain shift cannot be addressed effectively by increasing model complexity alone. Instead, explicitly analyzing distributional changes, identifying stable features, and incorporating limited target-domain knowledge into the learning process provide a more effective strategy for robust cross-domain intrusion detection. This revision additionally re-derived all reported results under a leakage-free protocol that computes drift statistics and draws few-shot adaptation samples exclusively from the CICIoMT2024 training split, and subjected the resulting model to KS-threshold, few-shot sample-size/seed, and feature-selection-baseline sensitivity checks, together with an exploratory secondary evaluation on a second target domain (CICAPT-IIoT2024). These checks confirmed the central operational claim—a reduction in FPR from 0.5469 to 0.0004—while refining its attribution: an additional Stable-only ablation configuration showed that drift-aware stable feature selection alone accounts for essentially the entire false-positive-rate reduction, whereas few-shot adaptation’s principal contribution is a reduction in false negatives. We regard this more precise, and independently verified, attribution of DEA-IDS’s two components as a strengthened rather than a diminished basis for the framework’s practical value.

A second revision pass further examined the framework at the level of individual attack families and against additional adaptation baselines. Per-attack-family analysis revealed that the reported false-positive-rate reduction, while dominant for high-volume flood-style attacks (DDoS/DoS, over 97% of attack traffic), coincides with a substantial loss of detection for three lower-volume attack families—ARP spoofing, malformed-MQTT traffic, and reconnaissance scanning—under stable feature selection. We report this trade-off explicitly rather than omit it, and identify a family-aware refinement of the stable feature selection criterion as a concrete priority for future work. Comparison against a target-only classifier and an unsupervised CORAL-based domain-adaptation baseline further showed that neither a small labeled target sample alone nor generic distributional alignment matches the operational reliability of the proposed drift-aware, few-shot approach, reinforcing the specific design choices made in DEA-IDS. Overall, DEA-IDS presents a data-centric framework that integrates explainability, drift analysis, and adaptation within a unified architecture. Under the evaluated IoT-to-IoMT cross-domain attack distribution, it delivers a substantial and independently verified improvement in false-alarm reliability and low-FPR discrimination, concentrated in the high-volume flood-style attacks that dominate this traffic mix, while detection of lower-volume attack families such as ARP spoofing, malformed-MQTT traffic, and reconnaissance scanning remains an open problem that the present stable-feature-selection criterion does not resolve (Section 6.7). We therefore present these results as evidence of a concrete, measurable improvement in operational reliability under the conditions tested, rather than as a general-purpose foundation for intrusion detection across arbitrary attack-family compositions or deployment settings.

## Figures and Tables

**Table 1:** Top Five Features with the Highest Distribution Shift (CICIoT2023 Training Split vs. CICIoMT2024 Training Pool).

Feature	KS Statistic	Wasserstein Distance
psh_flag_number	0.8876	0.4060
Srate	0.8058	1697.1957
Rate	0.8058	1697.1957
HTTPS	0.6509	0.6070
Duration	0.5751	43.2738

**Table 2:** KS-Threshold Sensitivity of the FewShot_Stable Configuration (CICIoMT2024 Test Set).

KS Threshold	# Features	F1-Score	FPR	ROC-AUC
0.05	17	0.9979	0.0008	0.9983
0.075	20	0.9979	0.0008	0.9983
0.10 (used in main results)	21	0.9944	0.0004	0.9984
0.125	22	0.9949	0.0004	0.9983
0.15	22	0.9949	0.0004	0.9983
0.20	22	0.9949	0.0004	0.9983

**Table 3:** Few-Shot Sample-Size and Seed Sensitivity (Mean ± Std, with Min–Max Range, over 10 Seeds, CICIoMT2024 Test Set).

# Adaptation Samples	FewShot F1	FewShot FPR (Mean ± Std; [Min, Max])	FewShot_Stable F1	FewShot_Stable FPR
10	0.9927 ± 0.0002	0.540 ± 0.020; [0.486, 0.553]	0.9942 ± 0.0002	0.0004 ± 0.0000
50	0.9938 ± 0.0014	0.448 ± 0.121; [0.255, 0.551]	0.9943 ± 0.0002	0.0004 ± 0.0000
100	0.9940 ± 0.0018	0.428 ± 0.149; [0.227, 0.554]	0.9943 ± 0.0000	0.0004 ± 0.0000
250	0.9961 ± 0.0008	0.248 ± 0.072; [0.172, 0.440]	0.9944 ± 0.0000	0.0004 ± 0.0000
500	0.9968 ± 0.0003	0.189 ± 0.029; [0.130, 0.232]	0.9947 ± 0.0007	0.0004 ± 0.0000
1000 (used in main results)	0.9970 ± 0.0003	0.161 ± 0.034; [0.098, 0.212]	0.9953 ± 0.0013	0.0004 ± 0.0000
2000	0.9975 ± 0.0003	0.106 ± 0.025; [0.056, 0.135]	0.9967 ± 0.0011	0.0004 ± 0.0000

**Table 4:** Cross-Domain Performance Comparison of Baseline and DEA-IDS.

Setup	Accuracy	Precision	Recall	F1-Score	ROC-AUC	FPR
Baseline	0.9855	0.9869	0.9986	0.9926	0.9963	0.5469
Stable-only	0.9882	1.0000	0.9880	0.9939	0.9983	0.0004
FewShot	0.9941	0.9960	0.9980	0.9970	0.9979	0.1660
FewShot_Stable	0.9892	1.0000	0.9890	0.9944	0.9984	0.0004

**Table 5:** Confusion-Matrix Counts on the CICIoMT2024 Test Set (N = 100,000; 2368 benign, 97,632 attack).

Setup	TN	FP	FN	TP	FNR
Baseline	1073	1295	152	97,480	0.0016
Stable-only	2367	1	1176	96,456	0.0120
FewShot	1975	393	200	97,432	0.0020
FewShot_Stable	2367	1	1077	96,555	0.0110

**Table 6:** MLP Benchmark Results under IID and Cross-Domain Conditions.

Scenario	Accuracy	Precision	Recall	F1-Score	ROC-AUC	FPR
IoT → IoT	0.9908	0.9964	0.9942	0.9953	0.9964	0.1430
IoT → IoMT	0.9905	0.9921	0.9981	0.9951	0.9443	0.3255

**Table 7:** Comparison with Directly Related Cross-Domain and Domain-Adaptation Studies.

Study	Source → Target	Method	Accuracy	F1-Score	AUC	FPR
Mahbub et al. (2026) [14]	ACIIoT2023 → CICIoMT2024	CWD + PSO + LR	Not reported	0.9423	Not reported	Not reported
Elangovan et al. (2026) [23]	Cross-dataset NIDS (2017–2024)	RF/GB/MLP/AE/1D-CNN	Not reported	0.69–0.78 (Macro)	Not reported	up to 0.30
Liu et al. (2024) [24]	Multi-domain IoT (auxiliary-domain transfer)	PointNet++-based transfer	0.964 (avg.)	Not reported	Not reported	Not reported
Vu et al. (2023) [25]	Cross-device IoT (Bot-IoT, 9 devices)	M2DA (AE-based DTL)	Not reported	Not reported	0.956 (best case)	Not reported (ROC-based)
DEA-IDS (this study)	CICIoT2023 → CICIoMT2024	FewShot + Stable	0.9892	0.9944	0.9984	0.0004

**Table 8:** Reported In-Distribution Results on CICIoMT2024 (No Cross-Domain Transfer).

Study	Method	Domain	Task	Accuracy
Shebl et al. [39]	DCNN	Same-domain	Binary/Multiclass	99.98%/99.86%
Benahmed et al. [40]	HBiLD-IDS (BiLSTM-DNN)	Same-domain	19-class	98.81%
Shaikh et al. [41]	HCLR-IDS (CNN + LSTM + RL)	Same-domain	Binary/18-class	99.58%/77.73%
Sharma and Shambharkar [42]	Multi-layered security architecture	Same-domain	Binary/Multiclass/Extended	99.49%/99.12%/98.56%
DEA-IDS (this study)	FewShot + Stable	Cross-domain	Binary (IoT → IoMT)	98.92% (F1 = 99.44%)

**Table 9:** Ablation Study Results on the CICIoMT2024 Test Set.

Setup	Accuracy	Precision	Recall	F1-Score	ROC-AUC	FPR
Baseline	0.9855	0.9869	0.9986	0.9926	0.9963	0.5469
Stable-only	0.9882	1.0000	0.9880	0.9939	0.9983	0.0004
FewShot	0.9941	0.9960	0.9980	0.9970	0.9979	0.1660
FewShot_Stable	0.9892	1.0000	0.9890	0.9944	0.9984	0.0004

**Table 10:** Example Stable Features Identified by Drift Analysis (KS < 0.1).

Feature	KS Statistic	Wasserstein Distance
Drate	0.0000	0.0000
ece_flag_number	0.0000	0.0000
rst_flag_number	0.0066	0.0011
fin_flag_number	0.0164	0.0018
HTTP	0.0151	0.0119

**Table 11:** Complete List of the 21 Stable Features (KS < 0.1), with Exact Drift Measurements.

Feature	KS Statistic	KS *p*-Value	Wasserstein Distance
SMTP	0.0000	1.0000	0.0000
DHCP	0.0000	1.0000	0.0000
IRC	0.0000	1.0000	0.0000
Drate	0.0000	1.0000	0.0000
Telnet	0.0000	1.0000	0.0000
ece_flag_number	0.0000	1.0000	0.0000
cwr_flag_number	0.0000	1.0000	0.0000
ICMP	0.0004	1.0000	0.0000
SSH	0.0004	1.0000	0.0000
rst_flag_number	0.0066	1.0000	0.0011
fin_count	0.0091	0.9999	0.0123
HTTP	0.0151	0.9276	0.0119
fin_flag_number	0.0164	0.8757	0.0018
syn_flag_number	0.0186	0.7616	0.0022
DNS	0.0228	0.5132	0.0041
Number	0.0254	0.3740	0.2042
Weight	0.0258	0.3568	5.2488
LLC	0.0560	0.0006	0.0174
IPv	0.0560	0.0006	0.0174
ARP	0.0565	0.0006	0.0178
Variance	0.0752	<0.0001	0.0338

**Table 12:** False Positive Rate Comparison under IoT → IoMT Evaluation.

Model/Configuration	FPR
MLP	0.3255
Baseline (XGBoost)	0.5469
Stable-only (XGBoost, no few-shot)	0.0004
FewShot	0.1660
DEA-IDS (FewShot_Stable)	0.0004

**Table 13:** Operational Robustness Comparison.

Model/Scenario	F1-Score	FPR
MLP (IoT → IoT)	0.9953	0.1430
MLP (IoT → IoMT)	0.9951	0.3255
DEA-IDS (IoT → IoMT)	0.9944	0.0004

**Table 14:** Comparison with Conventional Feature-Selection Baselines, Matched to 21 Features (CICIoMT2024 Test Set, FewShot Adaptation).

Feature-Selection Method	# Features	F1-Score	FPR
Drift-aware stable features (KS < 0.1, proposed)	21	0.9944	0.0004
Random selection (fixed seed)	21	0.9968	0.1660
Mutual information (top-21, source domain)	21	0.9969	0.1753
XGBoost feature importance (top-21, Baseline model)	21	0.9969	0.1731

**Table 15:** Exploratory Secondary Validation on CICAPT-IIoT2024 (IoT → Industrial IoT/APT); Interpret with Caution (Only 6 Attack Instances in the 100,000-Flow Test Sample).

Configuration	FPR (APT_Test)
Baseline	0.523
Stable-only	0.414
FewShot	0.406
FewShot_Stable	0.414

**Table 16:** Per-Attack-Family Recall (Detection Rate) on the CICIoMT2024 Test Set, by Ablation Configuration. The Benign row instead reports the within-family false-positive rate.

Family	N (Test)	Baseline	Stable-Only	FewShot	FewShot_Stable
Benign (FPR)	2368	0.5469	0.0004	0.1660	0.0004
ARP_Spoofing	96	0.7917	**0.0000**	0.7083	**0.0000**
MQTT-Malformed_Data	118	0.5000	0.0508	0.3559	0.1949
Recon	1685	0.9573	0.4332	0.9436	0.4813
MQTT-DDoS	3094	0.9997	0.9997	0.9997	0.9997
MQTT-DoS	754	1.0000	1.0000	1.0000	1.0000
TCP_IP-DDoS	66,116	1.0000	0.9999	1.0000	0.9999
TCP_IP-DoS	25,769	1.0000	0.9997	1.0000	0.9997
**Macro-Avg Recall (7 attack families)**	–	0.8927	0.6405	0.8582	0.6679
**Worst-Family Recall**	–	0.5000	0.0000	0.3559	0.0000

**Table 17:** Comparison with Additional Domain-Adaptation Baselines (CICIoMT2024 Test Set).

Configuration	Few-Shot Labels Used	Calibration Pool Used	F1-Score	FPR	FN	FP
Target-only (1000 samples, no source data)	Yes (1000)	No	0.9910	0.1394	1410	330
CORAL zero-shot (unsupervised alignment)	No	No	0.9882	0.9713	22	2300
FewShot (Section 5.2, for reference)	Yes (1000)	No	0.9970	0.1660	200	393
FewShot_Stable = DEA-IDS (Section 5.2, for reference)	Yes (1000)	Yes (up to 2745)	0.9944	0.0004	1077	1

**Table 18:** Extended, Imbalance-Robust Evaluation Metrics (CICIoMT2024 Test Set).

Setup	Balanced Accuracy	Macro-F1	MCC	PR-AUC
Baseline	0.7258	0.7950	0.6242	0.99991
Stable-only	0.9938	0.8974	0.8122	0.99995
FewShot	0.9160	0.9332	0.8673	0.99995
FewShot_Stable	0.9943	0.9045	0.8243	0.99995

**Table 19:** 95% Wilson-Score Confidence Intervals for FPR (CICIoMT2024 Test Set, N_Benign_ = 2368).

Setup	FP	FPR	95% CI Lower	95% CI Upper
Baseline	1295	0.5469	0.5268	0.5668
Stable-only	1	0.0004	0.0001	0.0024
FewShot	393	0.1660	0.1515	0.1815
FewShot_Stable	1	0.0004	0.0001	0.0024

**Table 20:** Comparison with Additional Drift Criteria and Generic Feature-Selection Methods, Matched to 21 Features/Components (CICIoMT2024 Test Set, FewShot Adaptation).

Method	# Features	Overlap with KS-Stable Set	F1-Score	FPR
KS-based (proposed, from Section 5.2)	21	21/21	0.9944	0.0004
Combined KS + Wasserstein (normalized rank)	21	21/21	0.9944	0.0004
Wasserstein distance alone (raw, unscaled)	21	19/21	0.9928	0.1263
ANOVA F-test (top-21)	21	7/21	0.9938	0.0790
RFE (logistic regression, top-21)	21	5/21	0.9970	0.1562
PCA (21 components; not directly comparable features)	21	n/a	0.9967	0.1474
SHAP-based selection (top-21 by mean |SHAP|, Baseline XGBoost)	21	3/21	0.9968	0.1782

**Table 21:** Random Forest Baseline Comparison (CICIoMT2024 Test Set).

Configuration	Classifier	F1-Score	FPR
Baseline	Random Forest	0.9862	0.6149
FewShot_Stable	Random Forest	0.9982	0.0156
Baseline	XGBoost (Section 5.2)	0.9926	0.5469
FewShot_Stable	XGBoost (Section 5.2)	0.9944	0.0004

**Table 22:** Re-Implementation of Mahbub et al. (2026)’s [14] CWD + LR Domain-Adaptation Method, Applied Within Our Leak-Free Protocol (CICIoMT2024 Test Set).

Configuration	F1-Score	FPR	FN/FP
Logistic Regression, no adaptation (LR_Only_NoCWD)	0.9946	0.4253	56/1007
Mahbub CWD-aligned LR, source-only (Mahbub_CWD_LR)	0.9940	0.4954	5/1173
Mahbub CWD-aligned LR + few-shot (Mahbub_CWD_LR_FewShot)	0.9946	0.4430	4/1049
DEA-IDS (FewShot_Stable, XGBoost; Section 5.2, for reference)	0.9944	0.0004	1077/1

**Table 23:** Nearest-Class-Mean (Prototypical) Few-Shot Classifier, Trained Only on the 1000-Sample Labeled Target Set (CICIoMT2024 Test Set).

Configuration	F1-Score	FPR	FN/FP
Prototypical few-shot classifier (no source data; this section)	0.9905	0.1068	1581/253
Target-only classifier (Section 5.14, for reference)	0.9910	0.1394	1410/330
DEA-IDS (FewShot_Stable, XGBoost; Section 5.2, for reference)	0.9944	0.0004	1077/1

**Table 24:** True Positive Rate (Recall) at Matched False Positive Rate Operating Points, Obtained from the ROC Curve on the CICIoMT2024 Test Set.

Configuration	Target FPR	Achieved FPR	TPR at That FPR
Baseline	0.1%	0.084%	0.941
Baseline	1%	0.971%	0.948
Baseline	5%	2.914%	0.986
FewShot_Stable	0.1%	0.042%	0.995
FewShot_Stable	1%	0.338%	0.996
FewShot_Stable	5%	3.000%	0.997

**Table 25:** Effect of the Number of Trusted Benign Target-Domain Calibration Rows on Stable-Feature Selection (CICIoMT2024 Test Set, FewShot_Stable).

Benign Calibration Rows	# Stable Features	Overlap with Main 21	F1-Score	FPR
50	21	21/21	0.9960	0.0017
100	19	18/21	0.7300	0.0198
250	22	21/21	0.9948	0.0004
500	21	21/21	0.9959	0.0004
1000	21	21/21	0.9958	0.0004
2745 (main result)	21	21/21	0.9944	0.0004

**Table 26:** Robustness of Stable-Feature Selection to Label Contamination of the Benign Calibration Sample (CICIoMT2024 Test Set, FewShot_Stable).

Contamination Rate	# Stable Features	F1-Score	FPR
0% (oracle-labeled)	21	0.9944	0.0004
1%	21	0.9944	0.0004
5%	21	0.9944	0.0004
10%	20	0.9979	0.0008

**Table 27:** Robustness of Stable Feature Selection and FewShot_Stable Performance to Independent Resampling of the 100,000-Row Source and Target Training Pools (CICIoMT2024 Test Set).

Pool Seed	# Stable Features	Overlap with Main 21	F1-Score	FPR
42 (main result)	21	21/21	0.9944	0.0004
7	21	21/21	0.9946	0.0004
123	21	20/21	0.9980	0.0004
2024	21	21/21	0.9930	0.0000
999	21	21/21	0.9964	0.0008
Mean ± Std	21 ± 0	20.8/21	0.9953 ± 0.0019	0.00042 ± 0.00030

## Data Availability

The datasets analyzed in this study are publicly available. The CICIoT2023 dataset is available at https://www.kaggle.com/datasets/himadri07/ciciot2023/data, and the CICIoMT2024 dataset is available at https://www.kaggle.com/datasets/limamateus/cic-iomt-2024-wifi-mqtt (both accessed on 6 April 2026).

## Abbreviations

The following abbreviations are used in this manuscript:

IoT	Internet of Things
IoMT	Internet of Medical Things
IDS	Intrusion Detection System
ML	Machine Learning
DL	Deep Learning
XAI	Explainable Artificial Intelligence
SHAP	SHapley Additive exPlanations
KS	Kolmogorov–Smirnov
FPR	False Positive Rate
FNR	False Negative Rate
MLP	Multi-Layer Perceptron
DEA-IDS	Drift-aware, Explainable and Adaptive Intrusion Detection System
ROC-AUC	Area Under the Receiver Operating Characteristic Curve
TP, TN, FP, FN	True Positive, True Negative, False Positive, False Negative
